# GraphAligner: rapid and versatile sequence-to-graph alignment

**DOI:** 10.1186/s13059-020-02157-2

**Published:** 2020-09-24

**Authors:** Mikko Rautiainen, Tobias Marschall

**Affiliations:** 1 Center for Bioinformatics, Saarland University, Saarland Informatics Campus E2.1, Saarbrücken, 66123 Germany; 2 Max Planck Institute for Informatics, Saarland Informatics Campus E1.4, Saarbrücken, 66123 Germany; 3 Saarbrücken Graduate School for Computer Science, Saarland Informatics Campus E1.3, Saarbrücken, 66123 Germany; 4 Heinrich Heine University Düsseldorf, Medical Faculty, Institute for Medical Biometry and Bioinformatics, Moorenstraße 5, Düsseldorf, 40225 Germany

**Keywords:** Genome graphs, Sequence alignment, Pangenome, Error correction, Long reads

## Abstract

*Genome graphs* can represent genetic variation and sequence uncertainty. Aligning sequences to genome graphs is key to many applications, including error correction, genome assembly, and genotyping of variants in a pangenome graph. Yet, so far, this step is often prohibitively slow. We present GraphAligner, a tool for aligning long reads to genome graphs. Compared to the state-of-the-art tools, GraphAligner is 13x faster and uses 3x less memory. When employing GraphAligner for error correction, we find it to be more than twice as accurate and over 12x faster than extant tools.Availability: Package manager: https://anaconda.org/bioconda/graphalignerand source code: https://github.com/maickrau/GraphAligner

## Background

Graphs provide a natural way of expressing variation or uncertainty in a genome [1, 2]. They have been used for diverse applications such as genome assembly [3–5], error correction [6–8], short tandem repeat genotyping [9], structural variation genotyping [10], and reference-free haplotype reconstruction [11]. With the growing usage of graphs, methods for handling graphs efficiently are becoming a crucial requirement for many applications.

Sequence alignment is one of the most fundamental operations in bioinformatics and necessary for a wide range of analyses. Aligning a sequence to a sequence is a well-studied problem with many highly optimized tools [12–15]. In contrast, aligning sequences to graphs is a newer field and practical tools only start to emerge, where most of the existing tools are specialized for one purpose such as error correction [6–8], or hybrid genome assembly [4]. The VG toolkit [16] provides a set of general-purpose tools to work with genome graphs. Although VG is capable of mapping long reads to graphs, it was tuned for aligning short reads, leading to slow runtimes for long read alignment. In summary, there is presently a lack of general-purpose tools for aligning long third-generation sequencing reads to graphs. Given the wide range of applications, including sequence assembly, error correction, and variant calling, and the steep decline in prices for long read sequencing, closing this gap is critical.

Outside of the bioinformatics community, an algorithm for aligning sequences to an arbitrary graph with unit costs was already discovered in 2000 in the context of hypertext searching by Navarro [17]. An important property of Navarro’s algorithm is that the runtime depends only on the number of nodes and edges and the length of the query sequence. Thus, complex cyclic graphs are (asymptotically) just as easy as simple linear graphs of the same size. Recently, it was proven that the runtime of Navarro’s algorithm is in fact optimal unless the strong exponential time hypothesis is false [18]. In 2002, *partial order alignment* [19] (POA), a special case of Navarro’s algorithm for acyclic graphs, was published for multiple sequence alignment. Although POA is defined only for acyclic graphs, it can be extended to cyclic graphs by unfolding cyclic components, which is the approach taken by the VG toolkit [16] and ExpansionHunter [9]. The practical efficiency of this unfolding depends on the read length, and the graph topology and complex cyclic areas can lead to very large unfolded graphs [20]. V-Align [20] aligns to cyclic graphs, but its runtime depends on the graph’s feedback vertex set size. Some tools use heuristic approaches for aligning to de Bruijn graphs using depth-first search [6, 8, 21]. Navarro’s algorithm has recently been generalized to arbitrary costs as well [22]. Our previous work [23] combined Navarro’s graph alignment algorithm with Myers’ bit-parallel algorithm [24], leading to speedups in practice between 5x-20x, but this algorithm is designed to compute the full dynamic programming table, making it unsuitable for aligning many reads to a large reference graph.

In contrast, most practical tools use a *seed-and-extend* strategy. Seeding depends on finding matches between the read and the graph and necessitates indexing the graph in some manner. Although asymptotically optimal algorithms for graph alignment are known, the lower bound for indexing a graph is currently unknown. *K*-mer-based indices have been used in many de Bruijn graph alignment tools [6, 21, 25]. The Positional Burrows-Wheeler transform [26] is a method for indexing multiple sequence alignments between genomes, which can be viewed as a special class of graph genomes. Indexing variation graphs is challenging because the number of possible paths can be exponential in the number of variants encoded. Typical approaches to handle this problem are to index only some of the variation by limiting the indexed paths either heuristically [16, 27, 28] or by using panels of known haplotypes [29, 30]. A recent method avoids the exponential blowup by dynamically indexing the graph and the reads, thereby exploiting that there can be exponentially many paths in the graphs, but not in the set of reads to be queried [31].

**Contributions** Here, we provide the first algorithm for *banded* sequence-to-graph alignment that scales to align noisy long reads to de Bruijn graphs of whole human genomes. We also apply a simple minimizer [32]-based seeding method which exploits the fact that long reads almost always span simple areas of the genome, unlike short reads which are more prone to being entirely embedded within a variation-rich area.

We describe our sequence-to-graph long read alignment tool GraphAligner. GraphAligner is designed to work with arbitrary graphs instead of specializing for one type of graph. We compare GraphAligner to minimap2 [13] for linear alignment and to the vg toolkit [16] for aligning to variation graphs. To show how better alignment methods improve downstream applications, we present a pipeline for error-correcting long reads based on graph alignment, which we compare to existing methods based on the same principle. Although using a similar process as existing tools, the better alignment strategy leads to an order of magnitude speedup and error rates less than half of the current state-of-the-art for whole human genome data. As another application, we present a simple genotyping pipeline based on building a pangenome graph and aligning long reads to it.

## Results

### Comparison to linear aligners

Regular sequence-to-sequence alignment is a special case of sequence-to-graph alignment, where the graph consists of a linear chain of nodes. We compare GraphAligner to a well-optimized sequence-to-sequence aligner, minimap2 [13], in whole human genome read alignment. We simulated 20x coverage reads from the GRCh38 reference using pbsim [33] with default parameters. We filtered out reads shorter than 1000 bp and reads containing any non-ATCG characters. Then, we aligned the reads to the reference using both minimap2 and GraphAligner. Then, we evaluated the mapping accuracy. We adopt the criteria used in the minimap2 evaluation [13] and consider a read correctly mapped if its longest alignment overlaps at least 10% with the genomic position from where it was simulated.

Table 1 shows the results. GraphAligner and minimap2 both align approximately as accurately, with minimap2 aligning slightly more reads correctly (95.0% vs 95.1%). GraphAligner takes about 3 × the runtime of minimap2, which we consider to be a modest overhead for a tool able to handle graphs in comparison to a highly optimized sequence-to-sequence mapping tool. Note that minimap2 is faster than commonly used competing tools, such as BWA-MEM [14], by more than one order of magnitude [13].

**Table 1:** Results of the linear comparison experiment

Aligner	Reads correctly aligned	CPU-time (HH:mm:ss)	Peak memory (Gb)
minimap2	95.1%	44:26:58	20.0
GraphAligner	95.0%	127:16:34	72.1

Simulated reads were aligned to the GRCh38 reference genome with minimap2 and GraphAligner

### Aligning to a graph with variants

In this experiment, we evaluated the mapping accuracy to a graph with variants. We used the chromosome 22 reference (GRCh37) and all variants in the Thousand Genomes project phase 3 release [34]. We constructed a variation graph from the reference and the variants using vg [16], producing a graph of chromosome 22 with 2,212,133 variants, containing on average one variant every 15 base pairs in the non-telomeric regions (the *variant graph*). Then, we simulated reads of varying lengths from the chromosome 22 reference sequence (GRCh37) using pbsim [33] with the default CLR parameters and aligned them to the graph with GraphAligner. We consider a read correctly mapped if its longest alignment overlaps at least 10% with the genomic position from where it was simulated and evaluate the number of reads correctly aligned. We also aligned the same reads to the chromosome 22 reference without variants (the *linear graph*) with GraphAligner to differentiate between reads which could not be aligned due to variants and reads which could not be aligned due to other reasons such as short read lengths leading to missed seeds. In addition to the reads simulated from the reference, we also simulated reads from de novo diploid assembled chromosome 22 contigs of the individual HG00733 [35]. This was done to test alignment accuracy on reads with realistic variants.

**Fig. 1:**
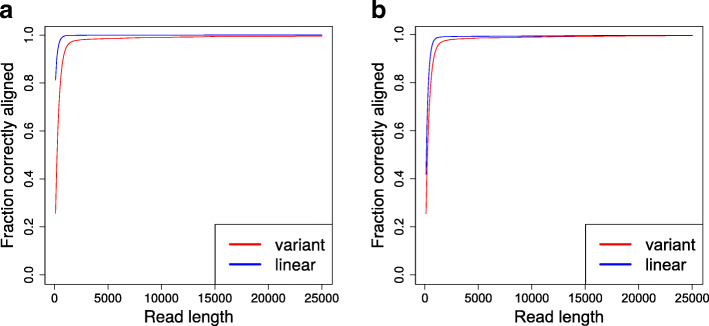
Fraction of reads correctly aligned at varying read lengths for the variant graph and the linear graph. Left: reads simulated from the GRCh37 reference. Right: reads simulated from de novo assembled contigs of HG00733

Figure 1 shows the results. The left part of the figure shows alignment accuracy for the reference simulated reads. For comparison purposes, the blue curve represents the results from mapping reads simulated from GRCh37 back to the (linear) reference genome and hence indicate the performance that can be achieved in an idealized setting. When aligning to the variant graph, 95% of the reference simulated reads are correctly aligned once read length grows above 1200 base pairs. At 1500 base pairs, 97.0% of the reads are correctly aligned to the variant graph. The right part of Fig. 1 shows the accuracy for reads simulated from de novo assembled contigs. Expectedly, the alignment accuracy for reads simulated from contigs is worse than for reads simulated from the reference (GRCh37) when aligning to the linear reference, but similar when aligning to the graph with variants. The results show that GraphAligner is capable of aligning long reads accurately to a variation-rich graph.

### Comparison to vg

In this experiment, we compared GraphAligner and vg [16] for aligning long reads. We used the graph from the previous experiment containing the chromosome 22 reference and all variants in the Thousand Genomes project phase 3 release [34]. We simulated reads from the chromosome 22 reference using pbsim [33] with default parameters. Then, we aligned the simulated reads to the graph using GraphAligner and vg.

Table 2 shows the results. GraphAligner aligned 96.6% of reads correctly, which is consistent with the results of the variation graph experiment. In contrast, vg aligned 93.8% of reads into the correct genomic region. However, we found that some of the alignments by vg were not consistent with graph topology, that is, the alignment traversed through nodes which are not connected by an edge. In some cases, the alignment “looped back” into the same reference area multiple times and even covered both alleles of a variant (Additional file 1: Figure S2). We did not evaluate how many of vg’s alignments were inconsistent with graph topology. GraphAligner’s runtime and peak memory includes both indexing and alignment. Despite including the indexing phase, we see that GraphAligner is almost ten times faster than vg’s mapping phase. When including vg’s indexing as well, GraphAligner is over thirteen times faster than vg. Peak memory use is three times smaller.

**Table 2:** Results of the comparison to vg

Aligner	Reads correctly aligned	CPU-time (HH:mm:ss)	Peak memory (Gb)
vg index	–	1:07:44	12.1
vg map	93.8%	3:13:15	4.1
GraphAligner	96.6%	0:19:30	3.6

Simulated reads were aligned to a chromosome 22 variation graph using both GraphAligner and vg

### Variant genotyping

We implemented a simple variant genotyping pipeline for long reads. First, a list of reference variants and a reference genome are used to build a pangenome graph using vg [16]. Then, long reads are aligned to the pangenome graph with GraphAligner. Finally, vg is used to genotype the variants according to the long read alignments.

We tested our variant genotyping pipeline using 35x coverage PacBio hifi reads from the individual HG002 [36], using the Genome in a Bottle (GIAB) benchmarking variant set version 3.3.2 for GRCh38 [37] as the ground truth. We tested three different scenarios: first, an ideal scenario where we use the variants in the GIAB variant set to build the graph; second, a more realistic scenario where we used variants from a different source, using the variant set by Lowy-Gallego et al. [38] called from the GRCh38 genome using the data from phase 3 of the Thousand Genomes Project (1000G) to build the graph; and third, using the variants from 1000G to build the graph but only evaluating the accuracy on variants which occur in both the 1000G and the GIAB variant set (1000G+GIAB). The reason for using the three different scenarios is that the genotyping pipeline cannot call novel variants; instead, it only genotypes variants which are already in the list of reference variants. This separates errors caused by the pangenome approach, and errors caused by imperfect reference variant set; the GIAB scenario will show how the pipeline would behave if the reference variant set was perfect, while the 1000G scenario will show the performance with a realistic, imperfect reference variant set and the 1000G+GIAB scenario will show the performance in a realistic setting for those variants that the pipeline could in principle genotype.

We evaluated the genotyping accuracy using RTG Tools vcfeval [39], which computes precision and recall for all variants, SNPs only and non-SNPs only. vg produces a confidence for each variant, and the evaluation produces a precision-recall curve for different confidence thresholds. We selected the threshold with the highest F-measure and report the precision and recall for that threshold. We evaluated the results in the Genome in a Bottle high confidence regions from all chromosomes in each scenario.

Table 3 shows the results. The genotyping accuracy is high in the GIAB scenario, but lower in the 1000G scenario. This shows that the choice of variant set affects the accuracy noticably with the F-measure dropping from 0.985 to 0.930. However, when excluding variants that the pipeline could not genotype even in principle, the F-measure is 0.970. This shows that a large part of the missing recall in the 1000G scenario is from variants that are not included in the reference variant set.

**Table 3:** Results of the variant genotyping experiment

Scenario	Variants	Precision	Recall	F-measure
GIAB	All	0.9929	0.9774	0.9851
	SNP	0.9994	0.9840	0.9916
	Non-SNP	0.9518	0.9353	0.9435
1000G	All	0.9694	0.8806	0.9229
	SNP	0.9806	0.9352	0.9574
	Non-SNP	0.8462	0.5417	0.6606
1000G+GIAB	All	0.9685	0.9712	0.9699
	SNP	0.9801	0.9797	0.9799
	Non-SNP	0.8556	0.8893	0.8721

Although previous publications [36] have shown performance exceeding the results in Table 3, the genotyping experiment shows an example use case for GraphAligner. The major limitation of the pipeline is that it cannot call novel variants, instead only genotyping known variants. We did not try varying the parameters of vg’s genotyping module or otherwise adjusting the genotyping process, which is tuned for short read genotyping and may not be optimal for long reads.

### Error correction

We have implemented a hybrid error correction pipeline based on sequence-to-graph alignment. Aligning reads to a de Bruijn graph (DBG) is a method of error correcting long reads from short reads [6, 7]. The idea is to build a DBG from the short reads and then find the best alignment between the long read and a path in the DBG. The sequence of the path can then be used as the corrected long read.

Zhang et al. [40] performed an evaluation of 16 different error correction methods. Based on their results, we chose FMLRC [8] as a fast and accurate hybrid error corrector for comparison. We also compare to LoRDEC [6] since our pipeline uses the same overall idea.

**Fig. 2:**
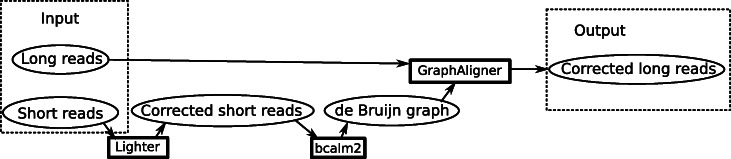
Overview of the error correction pipeline. The circles represent data and the rectangles programs

LoRDEC [6] builds a de Bruijn graph from the short reads, then aligns the long reads to it using a depth-first search and uses the path sequence as the corrected read. FMLRC [8] also aligns the reads to a graph, except instead of building one de Bruijn graph; it uses an FM-index which can represent all de Bruijn graphs and dynamically vary the *k*-mer size. FMLRC then corrects the reads in two passes, using different *k*-mer sizes. Our error correction pipeline is similar to LoRDEC. Figure 2 shows the pipeline. We first self-correct the Illumina reads using Lighter [41], then build the de Bruijn graph using BCalm2 [42], align the long reads using GraphAligner with default parameters, and finally extract the path as the corrected read.

Due to fluctuations and biases of Illumina coverage, some genomic areas are impossible to correct with short reads even in principle. Our pipeline has two modes: either we output the full reads, keeping uncorrected areas as is; or clipped reads, which remove the uncorrected areas and split the read into multiple corrected sub-reads, if needed. In the results, we present the full reads as “GraphAligner,” and the clipped reads as “GraphAligner-clip.” We similarly report “LoRDEC” as full reads and “LoRDEC-clip” as clipped reads. FMLRC does not offer an option to clip the reads, so we report only the full reads.

To evaluate the results, we use the evaluation methodology from Zhang et al. [40]. The long reads are first corrected, and then, the evaluation pipeline is run for both the raw reads and the corrected reads. The first step of the evaluation is removing reads shorter than 500 bp. Note that the reads are removed during the evaluation step, that is, they are corrected in the initial correction step and different reads may be removed in the uncorrected and corrected sets. After this, the remaining reads are aligned to the reference genome. The alignment yields several quality metrics, including number of aligned reads and base pairs, read N50, error rate, and genomic coverage. Here, we report error rate as given by samtools stats instead of alignment identity. Resource consumption is measured from CPU time and peak memory use. We use the *E. coli* Illumina+PacBio dataset (*E. coli*, called D1-P + D1-I by Zhang et al.) and the *D. melanogaster* Illumina+ONT dataset (Fruit fly, called D3-O + D3-I by Zhang et al.) from Zhang et al. [40]. In addition, we use whole human genome PacBio Sequel^1^ and Illumina^2^ data from HG00733, randomly subsampled to 15x coverage for PacBio and 30x for Illumina. We use the diploid assembly from [43] as the ground truth to evaluate against for HG00733. We did not include LoRDEC in the fruit fly or HG00733 experiments as the results in [40] show that FMLRC outperforms it in both speed and accuracy. Although we use the same evaluation method, our results are slightly different. This is due to two factors: First, Zhang et al. use LoRDEC version 0.8 with the default parameters, while we use version 0.9 with the parameters suggested for *E. coli* in the LoRDEC paper [6]. Second, Zhang et al. use FMLRC version 0.1.2 and construct the BWT with msBWT [44], while we use version 1.0.0 and construct the BWT with RopeBWT2 [45] as recommended by the FMLRC documentation.

Table 4 shows the results. The amount of aligned sequence is similar in all cases. For the PacBio data sets, the amount of corrected sequence is lower than the uncorrected input sequence, while for ONT, the amount of corrected sequence increases during correction. This is consistent with the observation that insertion errors are more common than deletions in PacBio and vice versa for ONT [47]. The number of reads is noticeably higher, and the N50 is lower for the clipped modes for both LoRDEC and GraphAligner, showing that most reads contain uncorrected areas and clipping the reads reduces read contiguity. In addition, the fruit fly and human experiments show that clipping the reads significantly reduces the genome fraction covered by the reads. The clipping is more pronounced in the more complex genomes, with the reads in the whole human genome dataset being on average cut into four pieces, around 4% of the genome lost due to clipping and a large reduction in read N50. We see that GraphAligner is about 30x faster and 2.7x more accurate than LoRDEC for *E. coli*. GraphAligner is over four times faster than FMLRC in all datasets. When not clipping reads, GraphAligner’s error rate is slightly worse then FMLRC for *E. coli* (0.51% vs. 0.30%), but substantially better for *D. melanogaster* (1.2% vs. 2.3%) and human (3.4% vs. 7.1%). For the human genome HG00733, GraphAligner hence produces over two times better error rates while the runtime is over twelve times faster.

**Table 4:** Results of the error correction experiment

Dataset	Method	# Reads	Bases (Mbp)	Aligned reads (%)	Aligned bases (%)	N50 (bp)	Genome fraction (%)	Error rate (%)	CPU time (hh:mm:ss)	Peak memory (GB)
E. coli	Original	85460	748.0	97.0	92.0	13990	100	13.1237	-	-
PacBio	LoRDEC	85316	716.5	97.9	92.9	13484	100	1.3902	10:11:28	5.0
	LoRDEC-clip	129754	654.5	99.9	99.8	8206	100	0.0881	10:11:28	5.0
	FMRLC	85260	706.5	97.7	94.8	13364	100	0.3016	4:16:43	2.6
	GraphAligner	85271	710.7	97.7	93.9	13411	100	0.5057	0:23:08	5.8
	GraphAligner- clip	91909	673.9	99.9	99.8	12146	100	0.0240	0:23:08	5.8
Fruit fly	Original	642255	4609.5	84.4	82.5	11956	98.77	16.1650	-	-
ONT	FMRLC	641956	4646.9	89.6	85.1	12087	98.62	2.3250	65:17:52	9.2
	GraphAligner	640548	4653.7	90.7	85.6	12109	98.63	1.2433	15:12:30	11.9
	GraphAligner- clip	762073	4188.3	99.3	94.7	8698	97.86	0.7087	15:12:30	11.9
HG00733	Original	2394990	48801.0	95.6	92.8	33109	95.27	13.5384	-	-
PacBio	FMRLC	2392533	48229.9	98.3	92.7	32823	95.19	7.1210	2222:13:44	234.5
	GraphAligner	2390656	48216.2	98.1	94.6	32879	94.89	3.3510	174:54:13	76.7
	GraphAligner-clip	8252956	42292.0	99.8	98.3	7973	91.91	1.3503	174:54:13	76.7

Reads shorter than 500 base pairs are discarded. The remaining reads were aligned to the reference using minimap2 [13], and the statistics were given by samtools [46] stats, except N50 which is calculated by a script from Zhang et al. [40] and resource use which are measured by “/usr/bin/time -v”

Our pipeline is a large improvement in runtime over the state-of-the-art. The error rates are competitive for simpler genomes and significantly better for more complex genomes. We hypothesize that the two-pass method used by FMLRC can in principle enable better correction than a single *k*-mer size graph, but FMLRC’s performance with the larger genomes is limited by their alignment method, while GraphAligner can handle the more complex genomes. When using the clipped mode, that is, when only considering parts of the reads that have been corrected, the accuracy in the corrected areas can approach or exceed the accuracy of short reads. This emphasizes the value of this clipped mode to users. The main source of errors are in fact uncorrected areas without sufficient short read coverage.

## Discussion

We have presented GraphAligner, a tool for aligning long reads to sequence graphs. Although GraphAligner is designed for graphs, it can also align to trivial linear graphs. Despite being slower than the highly optimized minimap2 tool, it is still faster than widely used linear mappers such as bwa [14]. In non-trivial variation graphs, GraphAligner outperforms vg by a factor of 13 in runtime.

GraphAligner is presently geared towards aligning long reads, which was our focus due to the absence of methods for this. The results in “Aligning to a graph with variants” section show that although GraphAligner can accurately align long reads in graphs containing large amounts of variation, the current seeding strategy can systematically fail to handle short reads in variation-dense regions. However, the core algorithmic components of GraphAligner could likely be used to also align short reads. To this end, we plan to integrate GraphAligner with PSI [31], a novel seeding approach that we developed recently to facilitate efficient and full-sensitivity seed finding across node boundaries.

As sequence alignment is a very fundamental operation and long reads are rapidly becoming more affordable to produce, we anticipate that GraphAligner will be used widely and will improve the performance and runtime of many downstream applications. Here, we have shown one example of this with our error correction experiment, where our pipeline improves on the state of the art and enables correcting long reads in mammalian scale genomes to high accuracy. It would be possible to combine GraphAligner’s alignment with the FM-index-based graph as used by FMLRC, which might yield an error correction pipeline as fast as and more accurate than our current results, which is an interesting avenue for future developments. We have also shown an example use case of using GraphAligner and vg for genotyping with long reads. The genotyping pipeline uses the vg genotyping module which is tuned for short reads. Adjusting the genotyping method to optimize the performance for long reads might be an another interesting avenue for future development. Other applications such as graph-based hybrid genome assembly also align reads to a graph, either explicitly [4] or by reducing the problem to sequence-to-sequence alignment [5]. It is likely that improved alignment methods will lead to improved results here as well, and we are currently investigating this further. In recent work, GraphAligner has also been employed for mapping long-read RNA-seq data to splice graphs [48], highlighting the breadth of possible use cases. Lastly, GraphAligner might enable scaling the haplotype-resolved genome assembly method that we demonstrated for yeast genomes [11] to mammalian genomes.

## Conclusions

We have implemented the sequence-to-graph alignment tool GraphAligner. As genome graphs become more common, efficient methods for aligning reads to genome graphs become more important. GraphAligner is able to work with a wide range of graphs, including graphs with overlapping as well as non-overlapping node sequences, and accepts GFA as well as vg graph formats. GraphAligner is competitive with well-optimized linear aligners when aligning to a linear genome, and outperforms existing graph alignment tools 13x in runtime. We have implemented a long read error correction pipeline using GraphAligner and showed that the method outperforms the current state-of-the-art, with a more than 2x improvement in error rate and over 12x improvement in runtime for whole human genomes.

## Methods

**Fig. 3:**
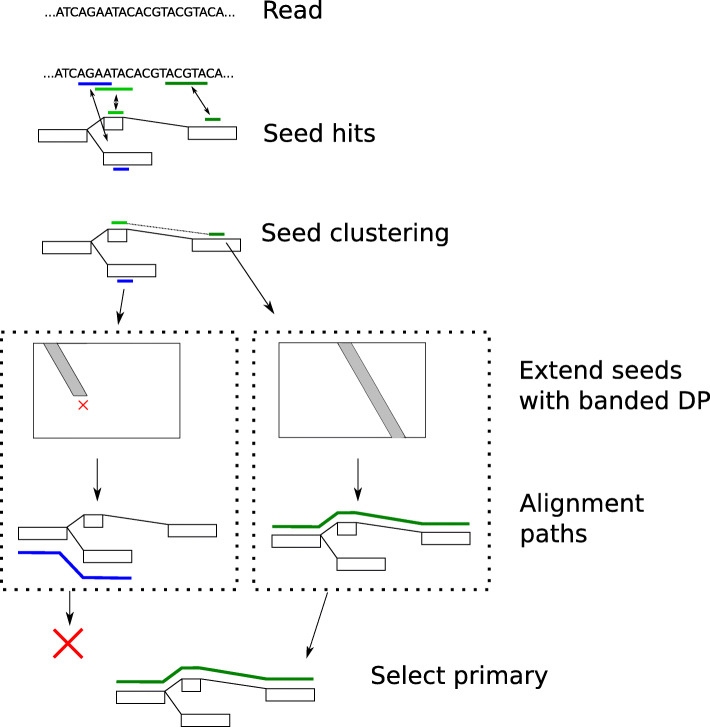
Overview of GraphAligner. Reads are aligned independently of the other reads. Seed hits are found by matching the sequence of the read to sequences inside nodes (small blue and green bars). Seed hits are clustered in locally acyclic parts of the graph and scored. Seed hits are then extended (small dotted boxes) with a banded dynamic programming algorithm, using Viterbi’s algorithm to decide when to clip the alignment (red X). Each seed hit can result in an alignment (blue and green paths). Alignments that overlap with an another, longer alignment in the query sequence are classified as secondary. Secondary alignments are discarded by default (red X) but can be included in the output with an optional parameter. The output is then written to a file either as alignments or corrected reads

Figure 3 shows an overview of GraphAligner. One IO thread reads sequences, which are passed to an arbitrary number of worker threads. Each worker thread aligns reads one at a time. The alignment algorithm uses a seed-and-extend method. Seeds are found by matching the read with the node sequences and then extended independently of each others with a bit-parallel banded dynamic programming algorithm. Finally, the primary and supplementary alignments are selected and passed to a second IO thread, which writes the results to a file.

### Data formats

We designed GraphAligner to use the most common file formats, and specifically be interoperable with vg [16] to leverage existing graph-based operations and pipelines. Graphs are inputted either in the binary vg graph format [16] or the human-readable graphical fragment assembly (gfa) format [49]. By allowing gfa, GraphAligner is moreover able to handle graphs with exact overlapping node labels, which is presently not supported by the vg file format, representing for example de Bruijn graphs. Reads are inputted in fasta or fastq and optionally gzip-compressed. Alignments are outputed in vg’s binary *gam* format, a generalization of SAM/BAM format [46] to graphs, and its equivalent text-based JSON format. Alignments can also be outputed in the GAF format [50], a generalization of minimap’s PAF format [49] to graphs.

### Graph model

GraphAligner inputs *bidirected graphs* [51, 52], which are capable of representing genome graphs commonly used in bioinformatics, including de Bruijn graphs [42, 53], assembly graphs [3, 54, 55], pangenomes [1], and variation graphs [2, 16]. Bidirected graphs model the double-stranded nature of DNA. The sequence is stored in the nodes, which can be traversed in two directions; either left to right (*forward*) with the node label or right to left (*backward*) with the reverse complement of the label. We notate a traversal’s *orientation* as + for the forward traversal and − for the backward traversal. The edges connect to either the *left end* or the *right end* of a node. A path through a bidirected graph enters a node from one end, traverses through the node, and then leaves via an edge in the opposite end. Formally, a bidirected graph can be defined as a $G_{b} = \{V_{b}, E_{b} \subseteq (V_{b} \times \{+, -\} \times V_{b} \times \{+, -\} \times \mathbb {N}), \sigma _{b}: V_{b} \rightarrow \Sigma ^{n}\}$, where *V*_*b*_ is the set of nodes, *E*_*b*_ contains a set of bidirected edges connecting ends of two nodes with an overlap, and *σ*_*b*_ is a function assigning a node label to each node in *V*_*b*_. We define the *opposite* of an orientation as $\bar {+} = -$ and $\bar {-} = +$. A bidirected edge (*v*_1_,*o*_1_,*v*_2_,*o*_2_,*n*) is equivalent to $(v_{2}, \bar {o_{2}}, v_{1}, \bar {o_{1}}, n)$, and we define that the set *E*_*b*_ contains both equivalent edges if the input graph contains either of them. We use the notation $\bar {s}$ to mark the reverse complement of a string *s*=*Σ*^*n*^.

The bidirected graph is first converted into a directed node-labeled graph which we call the *alignment graph*. The alignment graph is defined as a directed graph *G*_*a*_=(*V*_*a*_,*E*_*a*_⊆(*V*_*a*_×*V*_*a*_),*σ*_*a*_=*V*_*a*_→*Σ*^*n*^), where *V*_*a*_ is the set of nodes, *E*_*a*_ is a set of directed edges, and *σ*_*a*_ assigns a node label to each node in *V*_*a*_.

**Fig. 4:**
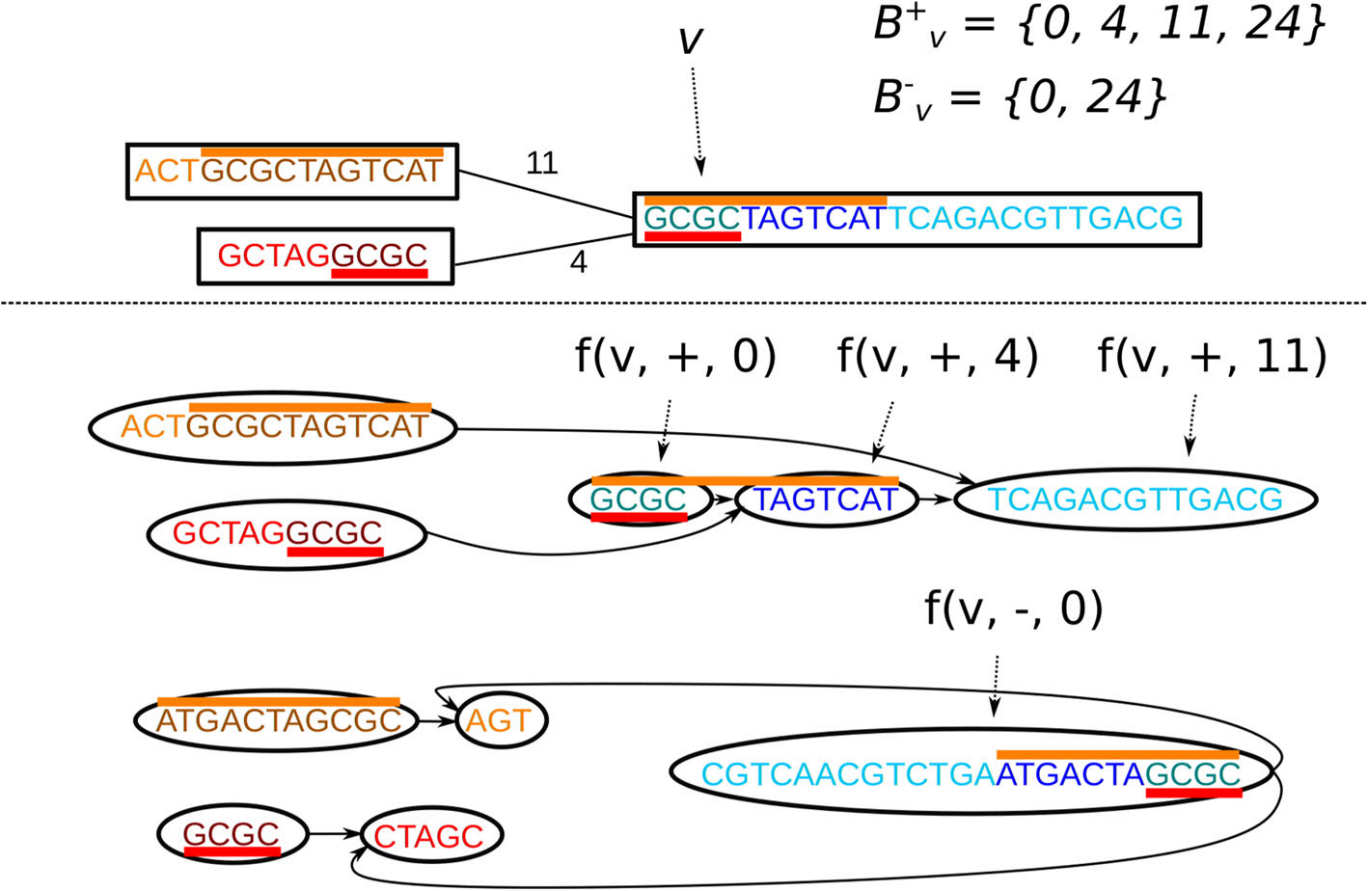
Converting a bidirected graph with variable edge overlaps to an alignment graph. Top: a bidirected graph with three nodes. The edges are labeled by their overlap. The red-colored bars represent the same sequence, which should not be duplicated during traversal. Similarly, the orange-colored bars represent the same sequence. Bottom: the alignment graph created from the top graph. The colors of the base pairs show how they match between the two graphs, with each sequence in the original graph represented by the same color in the alignment graph twice, once for the forward strand and once for the reverse complement. Similarly to the bidirected graph, the red and orange bars represent the same sequences. There are two subgraphs, one representing the forward traversal (top) and one the backward traversal (bottom) with reverse complemented node labels. Each edge introduces a breakpoint in the target node, splitting the node at the boundary of the overlap. The alignment graph then connects the ends of the overlap such that the overlapping sequence is only traversed once

The bidirected graph allows an overlap between edges, representing for example overlapping *k*−1-mers of a de Bruijn graph, or the read overlap in an assembly graph. Here, we consider the edges to be labeled by the number of overlapping nucleotides. When traversing via an edge with an overlap of *n* nucleotides, the path must skip the first *n* nucleotides of the target node. The overlaps can also vary between edges. Edge overlaps are handled by chopping the node into pieces at each overlap boundary. The alignment graph then has edges connecting the end of a node to the chopped boundary of the neighbor. This allows a path that ends at one node to enter the neighboring node without traversing the overlap twice. Figure 4 shows an example of the edge chopping for edges with variable overlaps.

Formally, given a bidirected node *v* and a set of incoming left edges *E*_+_={(*u*_1_,{+,−},*v*,+,*m*_1_),(*u*_2_,{+,−},*v*,+,*m*_2_),...}, we define a set of *forward breakpoints*$B^{+}_{v} = \{0, m_{1}, m_{2},..., |\sigma _{b}(v)|\}$, and given the set of incoming right edges *E*_−_={(*u*_1_,{+,−},*v*,−,*m*_1_),(*u*_2_,{+,−},*v*,−,*m*_2_),...} define a set of *backward breakpoints*$B^{-}_{v} = \{0, m_{1}, m_{2},..., |\sigma _{b}(v)|\}$. We also define a function $f: (V_{b}, o \in \{+, -\}, B^{o}_{v}) \rightarrow V_{a}$ which assigns each tuple of bidirected node, orientation, and breakpoint position (except |*σ*_*b*_(*v*)|) to one alignment graph node. Given the sorted sets of breakpoints, each successive pair of forward breakpoints $m, m' \in B^{+}_{v}$ causes a node to be inserted to the alignment graph with the label *σ*_*a*_(*f*(*v*,+,*m*))=*σ*_*b*_(*v*)[*m*,*m*^′^) representing the forward traversal, and each successive pair of backward breakpoints $m, m' \in V^{-}_{v}$ adds one node with the label $\sigma _{a}(f(v, -, m)) = \bar {\sigma _{b}(v)}[m, m')$ representing the backward traversal. We also add edges from *f*(*v*,+,*m*) to *f*(*v*,+,*m*^′^) and from *f*(*v*,−,*m*) to *f*(*v*,−,*m*^′^). Then, each bidirected edge *e*=(*v*_1_,*o*_1_,*v*_2_,*o*_2_,*m*) adds two edges to the alignment graph: one from *f*(*v*_1_,*o*_1_,|*σ*_*b*_(*v*_1_)|) to *f*(*v*_2_,*o*_2_,*m*) and another from $f(v_{2}, \bar {o_{2}}, |\sigma _{b}(v_{2})|)$ to $f(v_{1}, \bar {o_{1}}, m)$. In addition to the breakpoints added by the edges, we also add a breakpoint every 64 base pairs to each node because this makes it easier to encode the alignment graph node sequences using 64-bit words.

A node in the bidirected graph with *l* nucleotides adds $2 \lceil {l \over 64}\rceil $ nodes to the alignment graph, $\lceil {l \over 64}\rceil $ for the forward traversal, and $\lceil {l \over 64}\rceil $ for the backward traversal, and each edge can split up to two nodes and add up to four edges in the alignment graph. The number of nucleotides in the alignment graph is exactly twice the number of nucleotides in the bidirected graph. Therefore, the transformation produces an alignment graph whose size is within a constant factor of the bidirected graph.

During conversion, we also construct a *mapping* between the bidirected graph and the alignment graph. The mapping contains arrays *N*:*V*_*a*_→*V*_*b*_, describing for each node in the alignment graph which node in the bidirected graph it was created from; $O: V_{a} \rightarrow \mathbb {N}$ describing the alignment graph node’s offset within the bidirected node; and *D*:*V*_*a*_→{+,−} describing the orientation of the alignment graph node within the bidirected node. Using these arrays, we define a function $pos: (V_{a}, \mathbb {N}) \rightarrow (V_{b}, \mathbb {N}, \{+, -\})$ which maps each base pair (encoded as a node and offset) in the alignment graph to a base pair and orientation in the bidirected graph as

$$pos(v, o) = \left\{\begin{array}{ll} (N[v], O[v] + o, D[v]) & \text{if } D[v] = + \\ (N[v], |\sigma(N[v])| - (O[v] + o) - 1, D[v]) & \text{if } D[v] = - \end{array}\right. $$

Additionally, we store an array $A: V_{b} \rightarrow (V_{a}^{n}, V_{a}^{n})$, mapping each bidirected node to the pair of alignment graph nodes which represent its forward and backward traversals.

Taken together, the tables described above define a bijection between base pairs in the alignment graph, and combinations of a base pair and orientation in the bidirected graph, enabling positions to be unambiguously converted between the two graph representations. Given the two graphs and the mapping, GraphAligner aligns the read to the alignment graph and then converts the alignment back into the bidirected graph.

Both the read and the graph are allowed to contain ambiguous nucleotides (B, R, N, etc.) The alignment extension considers two ambiguous nucleotides a match if any of the possible nucleotides match; e.g., R (A or G) matches W (A or T) because both of them could be A, but R (A or G) does not match Y (C or T) because there is no overlap. Only the non-ambiguous characters A, T, C, and G are used for seeding.

### Seed hit finding

**Fig. 5:**
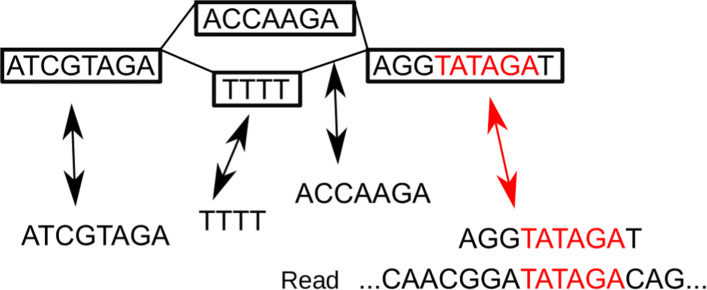
Seeding. Top: A graph with four nodes. Middle: The node sequences are extracted from the nodes. The arrows represent a mapping between the strings and nodes. Bottom: A read. Highlighted in red: Matches between the read and a string are converted into a match inside a node using the mapping

The first part of the seed-and-extend algorithm is finding seed hits. Here, we define seeds as exact matches between a read and a node sequence, but other definitions exist in the literature. Methods for finding exact matches between a read and paths in a graph have been developed [27–29, 31]. GraphAligner uses a simple method for transforming text matching in graphs to text matching in strings. Instead of matching reads to paths in the graph, reads are matched to node sequences in the graph. The nodes can be treated as a collection of strings which enables using efficient string matching algorithms. Reverse complement matches are also allowed. Figure 5 shows an example of matching a read to nodes in a graph. Note that we use the node sequences from the original bidirected graph, not from the directed alignment graph. The matching position is then converted from the bidirected graph to the alignment graph.

This approach finds only seed hits which are entirely contained in a node. For the special case of de Bruijn graphs, we hence find hits of length up to *k* due to the overlap between the nodes. However, in general, it misses seeds which cross a node boundary. As the results of the experiment in “Aligning to a graph with variants” section show, this is not an important limitation for long reads in practice, because long reads almost always touch linear parts of the graph, which usually leads to at least one seed hit.

GraphAligner’s default method for finding matches is by using *minimizers* [32]. A window of *w* base pairs is slid through the text and the smallest *k*-mers of each window according to a hash function are picked as the minimizers.

**Fig. 6:**
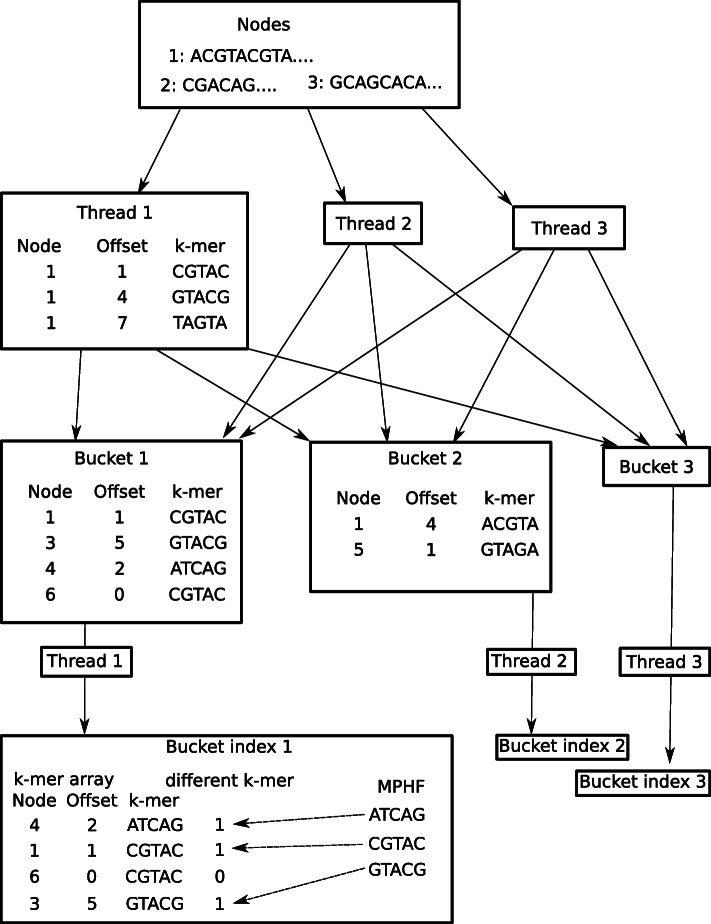
Building a minimizer index from a graph. Only the nodes of the graph are considered when building the index, and edges are ignored. Each node has an ID and a sequence. At the start, all nodes are labeled as *unprocessed*. Threads pick nodes one at a time from the pool of unprocessed nodes, and find minimizers in the node sequence. Then, the threads distribute the minimizers into buckets according to the modulo of their *k*-mer. Once all nodes have been processed, the threads proceed to index the buckets. Each thread picks one bucket and indexes it into a bucket index. The bucket index contains an array of the minimizers in that bucket sorted by the *k*-mer, a bitvector representing indices where a *k*-mer is different from the previous one, and a minimal perfect hash function which assigns each *k*-mer to the rank of the bit which represents the first instance of that *k*-mer in the sorted array

Building the minimizer index from a graph is multithreaded. Given *n* threads, each thread picks nodes one at a time and finds the minimizers in that node. The minimizers store the *k*-mer, the node ID, and the position within the node. The threads divide the minimizers into *n* buckets, implemented as parallel queues, based on the modulo of their *k*-mer. After all nodes have been processed, each thread picks one bucket and builds a bucket index from it. The minimizers in the bucket are first sorted based on their *k*-mer. Then, a bitvector representing different *k*-mers is built. The bitvector is set at indices where the current *k*-mer is different from the previous *k*-mer. A rank-select structure is built from the bitvector. Then, a minimal perfect hash function [56] is built to assign each *k*-mers to the rank of the first *k*-mer in the bitvector. Figure 6 shows the pipeline for indexing a graph. The most frequent fraction *x* of minimizers is not used for seeding, with default *x*=0.02*%*.

To query a *k*-mer, first the appropriate bucket index is found using the modulo of the *k*-mer. Then, the minimal perfect hash function is used to query the rank of the *k*-mer. The rank-select structure is then used to find the index in the sorted array where the *k*-mer is stored. Since the minimal perfect hash function can produce false positive hits for *k*-mers which were not used in constructing it [56], existence of the *k*-mer is verified by checking that the stored *k*-mer is equal to the queried *k*-mer. The number of occurrences of the *k*-mer can be checked in constant time by querying the index of the next-ranked bit. To retrieve the positions, the sorted *k*-mer array is iterated at the appropriate range. The index is implemented with succinct data structures from the SDSL library [57] and minimal perfect hashing from BBHash [56].

When finding seed hits, first a maximum number of seeds is calculated using a *seed density* parameter *d*. All k-mers of the read are queried to find matches and their frequencies. Given a read of length *l* and the *seed density* parameter *d*, only the least frequent *ld* minimizer hits are kept. In case of ties, all minimizers with frequency equal to the *ld*’th minimizer are kept.

The default values use *k*=19,*w*=30,*d*=5. These values are tuned for aligning reads to de Bruijn graphs with *k*=63. We have noticed that good parameters for aligning reads to a de Bruijn graph lead to poor alignment quality on variation graphs, and good parameters on variation graphs lead to high runtimes on de Bruijn graphs without improving alignment quality. For variation graphs, we instead recommend the parameters *k*=15,*w*=20,*d*=10, which are the parameters used in the linear comparison experiment, variant graph experiment, and comparison to vg experiment. We have observed that the shorter *k*-mer size improves seed clustering in variation graphs, while providing no improvement in de Bruijn graphs. We hypothesize that this is due to the chains of bubbles in de Bruijn graphs being too short for seed clustering to provide large improvements. Even shorter *k*-mer sizes did not lead to improved alignment accuracy in variation graphs. Shorter *k*-mer sizes also increase runtime due to more seeds being processed.

In addition to the built-in seeding methods, seeds can be inputted from a file, allowing an arbitrary external method to be used for seeding. The seeds must then be provided in GAM format [16], containing a position in the read, a position in the graph, and a match length.

Finally, GraphAligner has a mode for aligning without seeds. In this case, the extension algorithm is initialized with the entire first row of the dynamic programming table being considered and then proceeding as usual (see later sections for details). In this way, the alignment algorithm would implicitly scan the whole graph. The runtime is dependent on the graph size, so this mode is only practical for graphs up to a few million base pairs in size.

### Seed hit clustering

Typical alignment approaches [13] *chain* seeds to find the approximate position of the alignments. For linear sequences, seed chaining is solved with the *co-linear chaining* problem that exploits the fact that calculating the distance between seeds in a linear sequence is trivial. However, for graphs, the distance between seeds can be ambiguous as there are multiple paths connecting the seeds, and finding the distance in a graph is computationally more expensive than in a linear sequence [58]. GraphAligner clusters seed hits within acyclic subgraphs called *chains of superbubbles*.

**Fig. 7:**
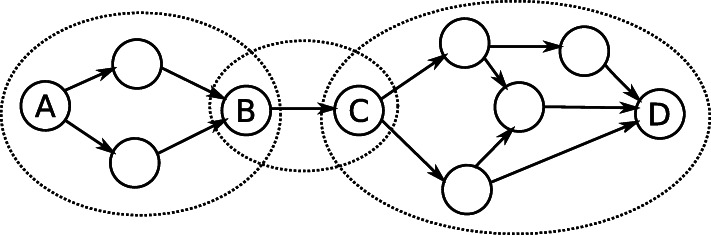
A chain of superbubbles containing three superbubbles. The solid circles are nodes connected by directed edges. The dashed circles show the three superbubbles. The three superbubbles start and end at A and B, B and C, and C and D, respectively. The three superbubbles form one chain of superbubbles since they share start and end nodes

A *superbubble* [59] is an induced acyclic subgraph with one unique entrance node, one unique exit node, and some amount, possibly zero, of *internal* nodes. All nodes in the superbubble are reachable from the entrance node, and all nodes reach the exit node. A superbubble must contain no edges from an internal node to a node outside of the superbubble or edges from outside the superbubble to an internal node. Superbubbles are defined only by the graph topology, and the node labels are irrelevant. Figure 7 shows examples of superbubbles. We use the algorithm from Onodera et al. [59] to detect superbubbles. Note that the superbubbles are found from the directed alignment graph, not from the original bidirected graph.

Given two superbubbles, we say that they belong in the same *chain* if the end node of one superbubble is also the start node of the other. Superbubbles may be chained this way to longer chains, and we say that they form a *chain of superbubbles*. In addition to superbubbles, we treat tips and small cycles as special cases that are included in the chain of superbubbles. An important property of a chain of superbubbles is that they induce an acyclic subgraph. The nodes can therefore be assigned linearized positions. GraphAligner arbitrarily picks one node in the chain of bubbles as the start node and then performs a breadth-first search along the chain to assign a linear position to each node. The pseudocode for the linearization is in Algorithm 1.


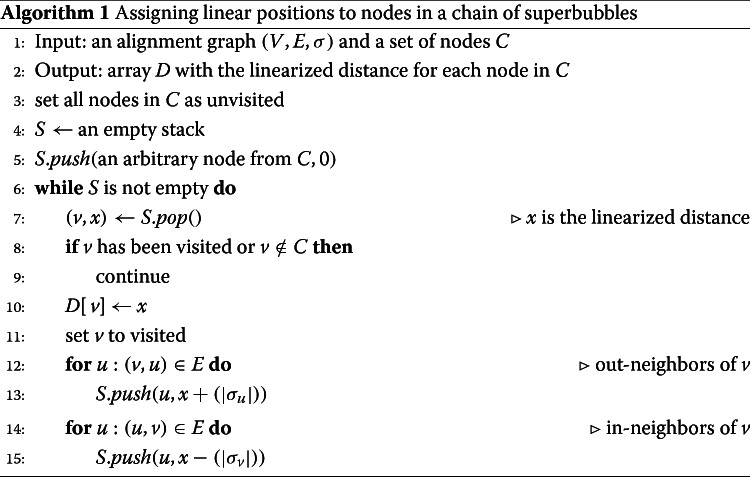


Given a chain of superbubbles, we can assign all seed hits in the chain a linear position. A position at offset *o* of node *v* is assigned a linearized position *D*[*v*]+*o*. Then, chaining algorithms for linear sequence alignment can be used for chaining the seed hits. We use the seed clustering algorithm from minimap [49], not to be confused with the seed chaining algorithm from minimap2 [13], to assign seed hits to clusters. Here, we briefly recap the seed clustering algorithm from minimap. Given a seed hit with position *r* in the read and a linearized position *b* in the chain of superbubbles, define the *diagonal position* of the seed hit as *d*=*r*−*b*. Then, two seeds in the same chain of superbubbles whose diagonal positions *d*_1_ and *d*_2_ are within a cutoff *c*=100, that is, |*d*_1_−*d*_2_|≤*c*, are connected together. The transitive closure of the connected seeds is the cluster.

Then, seed hits are scored according to their cluster size and uniqueness, with matches that occur fewer times in the graph weighted higher. Given a seed hit whose sequence occurs *x* times in the graph, and a maximum occurrence *m*, the unclustered *score* of the seed hit *i* is $s^{\prime }_{i} = m - x$. Then, given a cluster *C*, we calculate the number of base pairs in the read covered by at least one seed *c*_*C*_. The score of a seed hit that belongs in cluster *C* will then be *s*_*i*_=*si*′+*c*_*C*_.

The seed hits are ordered based on their clustered scores and extended from best scoring to worst scoring. Since the seed hits are not clustered arbitrarily across the graph, but only in simple subgraphs, the seed hit clustering is not used for limiting the paths explored or deciding when to end the alignment. The alignment algorithm used for the extension step instead decides which paths to explore and when to end the alignment (detailed in the “Extension”, “Bit-parallel operations”, “Banded alignment on graphs”, “Storing a partial DP matrix”, and “Partial alignments” sections). Seeds included in alignments from previously explored seeds are skipped.

Finally, a *seed extension densitye* parameter is used for choosing how many seed hits to extend. Given a read of length *l* and the extension density parameter *e*, seeds are extended starting from the highest scoring seed until *le* seed hits have been extended, with ties also extended. Seeds which are skipped due to being included in a previous alignment do not count against this limit. This filter is applied after the seed hits have been clustered and scored. The default values for *e* is *e*=0.002 for de Bruijn graphs and *e*=1 for variation graphs.

### Extension

GraphAligner uses a dynamic programming (DP) algorithm to extend the seeds. The starting point of the DP is the well known Needleman-Wunsch algorithm for sequence alignment [60]. This algorithm has been generalized to sequence-to-graph alignment by Navarro [17]. In a previous work [23], we further generalized Myers’ bit-parallel method [24] to sequence-to-graph alignment to improve the runtime.

In short, the algorithm calculates a *DP matrix* whose scores describe the edit distance of an alignment ending at a specific position in the read and a specific position in the graph. The calculation proceeds in a sliced manner, first calculating a horizontal *slice* of the topmost 64 rows, then calculating the next topmost slice and so on. For details on how to calculate the DP matrix for graphs in a bit-parallel manner, we refer the reader to [23]. In the following, focus on describing the extensions over this previous work that were necessary to make GraphAligner scale to large graphs: first, a faster algorithm for merging bitvectors; second, how to apply *banded alignment* [61] to graphs, reducing the area in the DP matrix which needs to be calculated and greatly reducing runtime and memory use; and third, how to efficiently store a partial DP matrix of a graph.

### Bit-parallel operations

The DP extension algorithm requires merging bitvectors at nodes with an in-degree of at least two. In our previous work [23], we described an *O*(log*w*) algorithm for merging two *w*-bit sized bitvectors. We have refined this operation further and created an algorithm which is much faster in practice but with a theoretically slower runtime of *O*(*w*). In practice, the *O*(*w*) algorithm takes on average around 50 instructions per merge, while the *O*(log*w*) algorithm takes on average around 300 instructions per merge for 64-bit bitvectors. The code and detailed explanation of the merging algorithm is Additional file 1: Section A.

### Banded alignment on graphs

In sequence-to-sequence alignment, banded alignment [61, 62] is a technique for speeding up the alignment while guaranteeing that the optimal alignment is still found as long as the number of errors is small. The idea is that given a start position of the alignment and a maximum edit distance, a diagonal parallelogram is selected, and the DP matrix is calculated only inside the parallelogram [62]. Formally, given a banding parameter *b* and a start column *p*, a cell at row *x* and column *y* is calculated if |*x*+*y*−*p*|≤*b*. The width of the parallelogram is 2*b*, and the optimal alignment is guaranteed to be found if it has at most *b* errors. The runtime of the alignment is now *O*(*nb*) where *n* is the length of the query sequence. The runtime no longer depends on the size of the reference, leading to a large speedup.

The parallelogram technique cannot be used in graphs due to the non-linear structure. At each fork, the parallelogram should continue to both paths. This would mean that the size of the band could grow very large, and the bookkeeping involved in tracking the band would introduce heavy overhead, possibly exponential to the size of the graph.

Recently, a dynamic banding approach was proposed for linear sequence alignment [63]. The approach allows the band to move during the alignment based on the scores of the alignment. The method requires calculating the DP matrix in an antidiagonal order, which cannot be easily extended to graph alignment since the antidiagonal is ambiguous for forks.

**Fig. 8:**
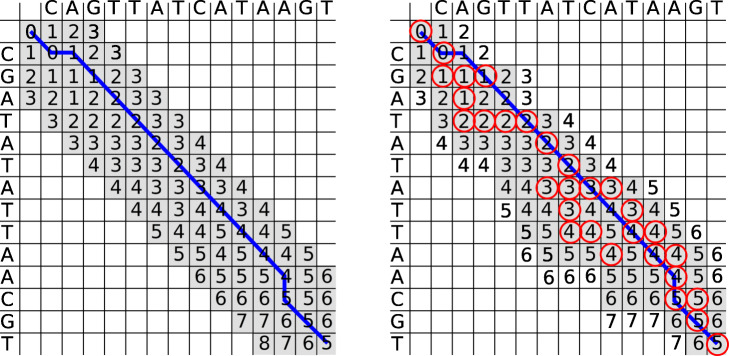
Left: regular banded alignment with *b*=3. The reference is on top and the query on the left. The gray cells are inside the band and are calculated. The blue line shows the traceback of the optimal alignment. Right: score based banding with *b*=1. The reference is on top and the query on the left. The gray cells are inside the band and the blue line is the traceback. The red-circled cells are the minimum for each row, which are discovered during the calculation of the matrix and define whether a cell is inside the band or not; a cell is inside the band if its score is within *b* of the minimum score in the same row. The cells with a number on a white background are calculated to discover the end of the band, but they are not inside the band and are ignored when calculating the next row. The band can wander around the DP matrix and change size, automatically spreading wider in high error areas and narrower in low error areas. Note that the score-based banding parameter is 1 in comparison to 3 in the regular banding to the left. The implementation uses a coarser band of 64 x 64 blocks instead of individual cells

Instead, we introduce a novel dynamic banding approach based on the scores in the DP matrix. The principle is that for each row, we find the minimum score *m* and define a cell to be inside the band if its score is at most *m*+*b*. This handles arbitrary graph topologies with very little bookkeeping and no special cases. Figure 8 shows an example. Since the band depends on the minimum score in a row, which is initially unknown, we do not initially know which parts of the DP matrix are included in the band. Instead, we “discover” the minimum score and the edges of the band as we calculate the DP matrix. To find the edge of the band, cells must be calculated until their score is higher than *m*+*b*, at which point the cell is out of band.

**Fig. 9:**
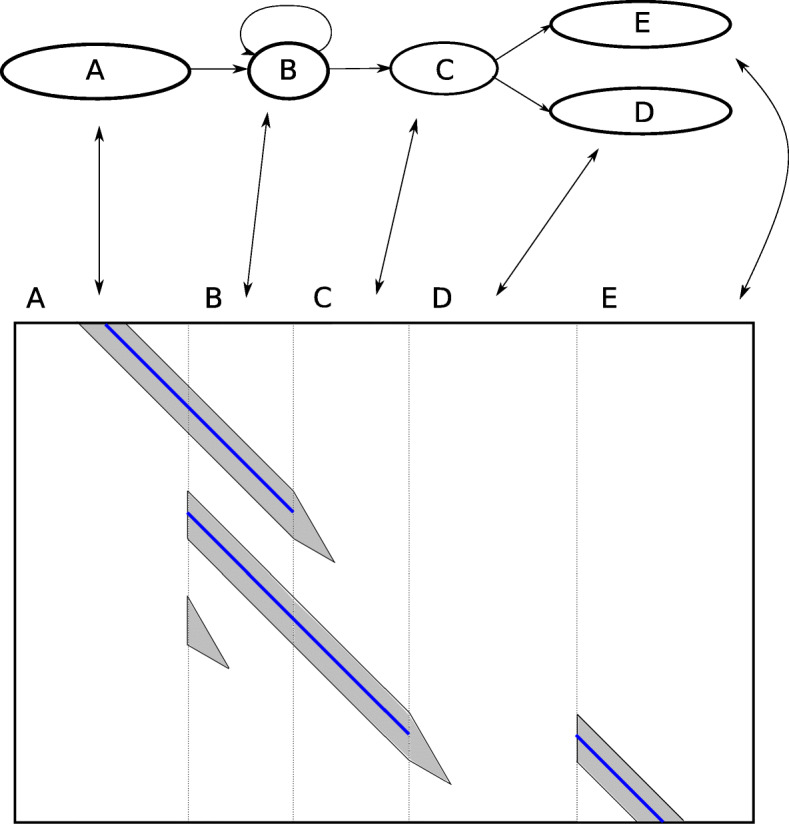
Dynamic score-based banding applied on a graph. Top: an alignment graph. Bottom: The DP matrix for aligning a read to the above graph. The arrows show the correspondence between nodes in the graph and columns in the DP matrix. The dotted lines separate the nodes. The gray area represents parts of the DP matrix which are calculated, and the parts in the white area are not calculated. At each fork, the band spreads to all out-neighbors. The score-based banding implicitly limits the exploration of the alternate paths; as the scores in the alternate paths become worse than the optimal path, the explored part shrinks until finally the exploration stops completely. The blue line shows the backtrace path

Figure 9 shows how the dynamic score-based banding handles different topological features. At each fork, the band spreads to all out-neighbors. This explores the different paths the alignment could take, while the score comparison implicitly limits how far the exploration proceeds.

The dynamic banding introduced here is not symmetric between the query and the reference. That is, when aligning two linear sequences, the band will be different depending on which sequence is the query and which is the reference. However, the sequence-to-graph mapping algorithm already introduces an asymmetry since the graph must be the reference.

**Fig. 10:**
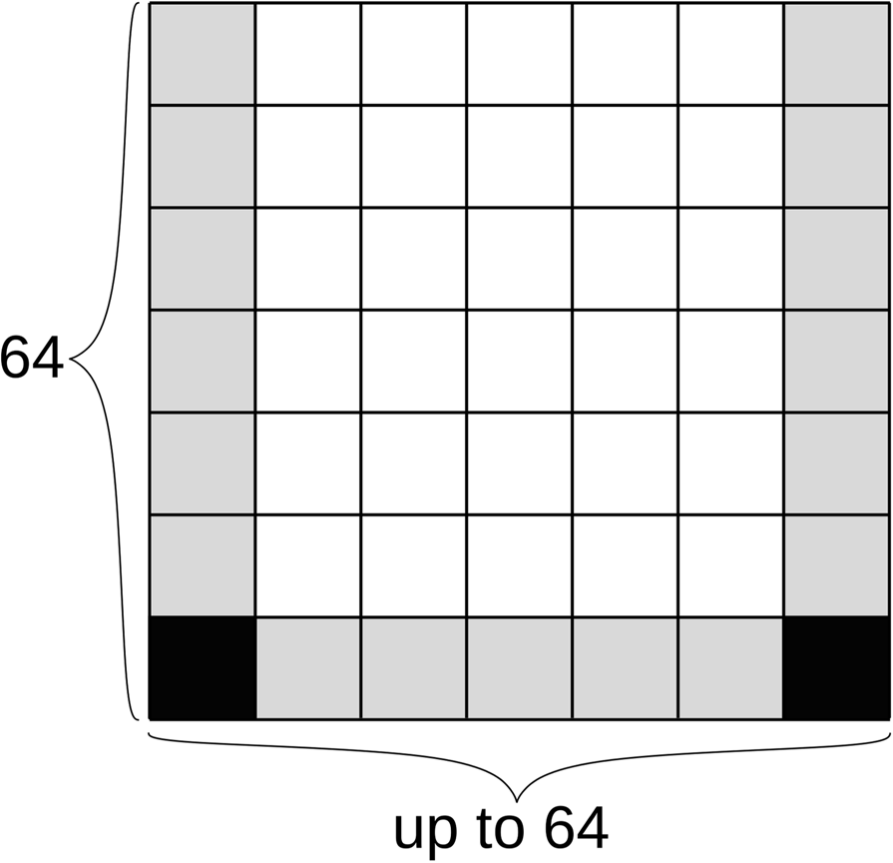
Sparse storage of the DP matrix. Each node is stored in blocks of 64 rows and up to 64 columns. The scores of the corner cells (solid black) are stored explicitly, using 4 bytes per cell. The border cells (gray) are stored with a score difference, using 2 bits per cell. The middle cells (white) are not stored

Due to the bitvector-based calculation, the implementation is slightly different from the theoretical description above. The band is defined over blocks in the DP matrix (see Fig. 10) instead of individual cells in the DP matrix. In addition, a block’s minimum score is compared to the minimum score in the last row of a 64-row slice. That is, for each 64-row slice, the minimum score *m* is found. Then, a block in the DP matrix is inside the band if the score of any cell in the block is at most *m*+*b*.

Since *b* represents a score difference, the score guarantee is now stronger than in the linear case. The optimal alignment is found as long as the optimal alignment’s score at any row is within *b* of the minimum score of that row. This trivially includes the case that the optimal alignment has *b* errors.

However, the size of the band is no longer bounded by *b*. This means that the score-based banding can lead to an impractically large band in certain cases. In an unrealistic extreme case, a fully connected graph will be entirely included in the band even with *b*=1 and regardless of the graph size. But also in practice, human whole genome de Bruijn graphs contain highly complex subgraphs (Additional file 1: Figure S1). In this example, including all cells with a low score difference will contain a very large part of the subgraph, increasing both runtime and memory use. To handle these cases, we introduce a second banding parameter, the *tangle effortC*. This determines how much effort the DP extension spends in tangled areas of the graph. As we calculate the DP matrix, we keep track of how many cells have been calculated in the current slice. Once this number grows above *C*, we stop calculating the current slice, keep the scores as they are, and move to the next slice. This bounds the runtime in tangled regions. However, this is a heuristic method which depends on the assumption that the correct path will be calculated before false positive paths.

In our previous work [23], we used the *minimum changed value* to decide the order in which we calculate the DP matrix. If the parameter *C* is not given, the DP extension uses the minimum changed value as described in the earlier work. However, if the parameter *C* is given, we use a different order, the *minimum changed priority value* of a cell to decide the order. We define the *priority value* of a cell based on the observed error rate of the best alignment so far. We calculate the observed error rate *e* based on the minimum score of the last calculated slice. Given a minimum score *s* at row *y*, the observed error rate is $s \over y$. With an error rate *e*, a DP cell at row *m* with a score of *k* has a priority value of ${k \over e} - m$, or 64*k*−*m* if $e \leq {1 \over 64}$. When recalculating a column, the *changed priority value* of a cell is the priority value of a cell in that column which changed, and the *minimum changed priority value* of a column is the minimum of the changed priority values. The intuition is that the priority value of a cell describes “how good” the alignment at a cell is; a value of 0 means as good as the best alignment so far, negative is better than that and positive worse than that. The minimum changed priority value is essentially a greedy heuristic for exploring the most promising paths first. The result is that the minimum changed priority value leads to a higher probability of correctly aligning through a tangle than the minimum changed value when the tangle effort is limited. Without a limit on the tangle effort, using the minimum changed priority value would lead to the scores eventually converging to the same values as the minimum changed value, but the worst-case runtime bounds are worse than for the minimum changed value.

### Storing a partial DP matrix

In sequence-to-sequence alignment, the banded DP matrix can efficiently be stored as a two-dimensional matrix with 2*b* diagonals, where *b* is the width of the band. However, in sequence-to-graph alignment, the banded matrix cannot be stored contiguously due to the non-linear nature of graphs. We conceptually treat the DP matrix as a sparse three-dimensional matrix, with one dimension for node ID, one for node offset, and one for read offset.

The implementation stores the DP matrix as a hash table from node IDs to a sparse representation of the alignment between a substring of the read and the sequence of a node. The sparse representation explicitly stores scores at the “bottom corners,” and the score differences between the left, right, and bottom “border cells.” Figure 10 shows an example of this. The middle cells are not stored at all. Instead, the explicitly stored cells allow recalculating the middle cells when needed. This only happens when recalculating cyclic areas, which requires recalculating the middle cells anyway, and during the backtrace, which requires recalculating only the path taken by the backtrace. The sparse representation requires 56 bytes per node, plus memory overhead from the hash table, while using the same data representation that the bit-parallel calculation uses and having no runtime overhead from compression or conversion between different formats. For comparison, the information theoretic lower bound for storing all cells in the DP matrix for one node with optimal compression is ${{\log _{2} {3^{64*64}}}\over {8}} \approx 812$ bytes and storing only the border cells is ${{\log _{2} {3^{64+64+62}}}\over {8}} \approx 38$ bytes.

### Partial alignments

Previously, software such as BLAST [12] have used the *X-drop* heuristic [64] to end alignment. In the X-drop heuristic, the algorithm keeps track of the highest alignment score seen so far. Once the scores within the current row to be calculated drop below a cutoff defined on the highest alignment score and a parameter, the alignment is ended and the cell with the highest alignment score is used to start the backtrace. The X-drop heuristic requires using local alignment with a scoring scheme where higher values are better alignments, and it is not trivial to correctly extend it to the unit costs required by the bit-parallel algorithm.

**Fig. 11:**
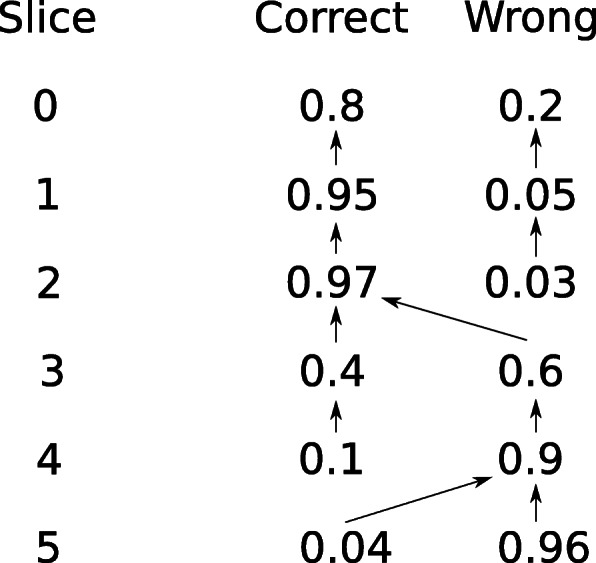
An example of using Viterbi’s algorithm for estimating correctness per slice. The error rates of the minimum alignment per slice (not shown in figure) are the observations. The numbers represent the probability of the alignment being in the specific state at the specific slice. The arrows represent the predecessor state for each state in each slice. Slice 2 is guaranteed correct since the predecessor for the wrong state in slice 3 is through the correct state. Similarly, slice 4 is guaranteed wrong since the predecessor for the correct state in slice 5 is through the wrong state. None of the other slices are guaranteed correct or wrong. The final backtrace will consider slices 0, 1, and 2 correctly aligned and slices 3 to 5 wrongly aligned, and only the sequence in slices 0–2 will be reported in the alignment

During alignment, we use Viterbi’s algorithm [65] to estimate the correctness at each slice boundary. That is, we seek to estimate the probability that the slice contains the correct alignment. The observations of the algorithm are the minimum scores at the end of each slice. Conceptually, we use a hidden Markov model with two hidden states, which are labeled *“correctly aligned”* and *“wrongly aligned”*. We model the emissions and transition probabilities such that the correctly aligned state outputs an error rate of 20% and the wrongly aligned an error rate of 50%. These error rates were selected empirically by aligning Oxford Nanopore (ONT) reads to either the correct or the wrong genomic position, using the assumption that the errors of reads to be processed are at most as high as for these ONT reads. The probabilities of the correct and wrong states and their predecessor states are calculated for each slice during alignment. After calculating slice *n*+1, we define slice *n* as *guaranteed correct* if the predecessor of the wrong state in slice *n*+1 is the correct state in slice *n*. The intuition behind this is that any alignment in slices *n*+1 and later, correct or wrong, must backtrace through the correct state at slice *n*, so the read is correctly aligned at least until that point. We also similarly define a slice *n* as *guaranteed wrong* when predecessor for the correct state in slice *n*+1 is the wrong state in slice *n*. Figure 11 shows an example of this.

We use the correctness estimate to vary the banding parameters. We use two parameters, an initial banding parameter *b* and a ramp banding parameter *B*>*b*. Once the probability of the wrongly aligned state is higher than the probability of the correctly aligned state, we backtrace to the last guaranteed correct slice, switch to the higher ramp banding parameter, and re-align until we have reached the original slice. Note that this is a looser condition than reaching a guaranteed wrong slice.

We also use the Viterbi estimate to end the alignment. Once we have reached a guaranteed wrong slice, the extension can no longer produce anything useful. In this case, we backtrace to the last correct slice and return the partial alignment of the read up to that position.

After extending the seed hits, we are left with a list of partial alignments. We then select a non-overlapping subset of primary and supplementary alignments in a heuristic manner. We greedily pick alignments from longest to shortest and include an alignment as long as it does not overlap with a previously picked alignment. The primary and supplementary alignments are then written as output. The overlapping alignments are considered secondary and discarded by default, with an optional switch to output secondary alignments as well.

### Experimental setup

All experiments were ran on a computing server with 48 Intel(R) Xeon(R) E7-8857 v2 CPUs and 1.5Tb of RAM. Every program was given 40 threads in the command line invocation. Runtime and memory use was measured with “/usr/bin/time -v” in all experiments.

In the linear comparison experiment, we ran minimap2 with the command “minimap2 -x map-pb -a -t 40,” corresponding to the recommended parameters for PacBio reads and 40 threads. We ran GraphAligner with “GraphAligner -t 40 -x vg,” using 40 threads and our recommended parameters for variation graphs. We used minimap2 version 2.17-r941 and GraphAligner version 1.0.11. Reads were considered to be correctly aligned if the position of their longest alignment overlapped at least 10% with the genomic position from where the read was simulated.

In the variant graph experiment, we constructed the graph with the command “vg construct -a -r chr22.fa -v chr22.vcf -p -t 40 -m 3000000.” We ran GraphAligner with the command “GraphAligner -t 40 -x vg,” using 40 threads and our recommended parameters for variation graphs. We used GraphAligner version 1.0.11. The genomic interval of the alignment was calculated only from the parts of the alignment which covered a reference node. That is, parts of the alignment path which corresponded to a non-reference variant were ignored, and the reference interval was taken as the minimum and maximum of the reference positions covered by the alignment. Reads were considered to be correctly aligned if the position of their longest alignment’s genomic interval overlapped at least 10% with the genomic position from where the read was simulated.

To evaluate the alignment accuracy of the reads simulated from de novo assembled contigs, we lifted over their coordinates to the GRCh37 reference chromosome 22 using minimap2 [13]. We first aligned the assembled contigs to the reference with minimap2, then used “paftools.js liftover” from minimap2 to lift over the coordinates of the simulated reads from the contigs to the reference. We evaluated alignment accuracy only for the reads which could be lifted over. The de novo assembled contigs were separated by haplotype, and results were evaluated separately per haplotype. However, the right side of Fig. 1 shows the average results across both haplotypes. The average was plotted instead of separate haplotypes because the results for individual haplotypes differed at most 0.3% and the curves could not be distinguished visually.

In the vg comparison experiment, we used the graph from the variant graph experiment. We ran GraphAligner with the command “GraphAligner -t 40 -x vg,” using 40 threads and our recommended parameters for variation graphs. For vg, we first preprocessed the graph as suggested by vg documentation with the commands “vg mod -X 256” and “vg prune.” The runtime of the preprocessing was not included in the results. We indexed the graph with the command “vg index -t 40 -x chr22.xg -g chr22.gcsa” using 40 threads to construct the indices required for mapping. We mapped the reads with the command “vg map -t 40 -m long,” using 40 threads and parameters for long read alignment. We used vg version 1.23.0. Read alignment accuracy was evaluated the same way as in the variation graph experiment. Note that the evaluation method only distinguished whether the read alignment overlapped with the correct genomic interval and does not evaluate the correctness otherwise. In particular, alignments whose path in the graph is not consistent with graph topology, such as aligning to both branches of a SNP (Additional file 1: Figure S2), could still be counted as “correctly” aligned.

In the genotyping experiment, we constructed the pangenome graph using vg with the command “vg construct -a -r reference.fa -v variants.vcf -p -t 40 -m 30000000” and detected snarls using the command “vg snarls graph.vg > graph.snarls.” We aligned the reads using GraphAligner with the command “GraphAligner -t 40 -x vg,” using 40 threads and our recommended parameters for variation graphs. We genotyped the sample by first running “vg pack -x graph.vg -g alignments.gam -o alignments.pack,” and then “vg call graph.vg -k alignments.pack -r graph.snarls -v variants.vcf -s HG002”, using the default genotyping method with the default parameters. We used the Genome in a Bottle callset version 3.3.2 for GRCh38. We used the version 2a (release 20190312) of the variant set from Lowy-Gallego et al. [38]. We used vg version 1.23.0 and GraphAligner version 1.0.11.

## Supplementary information

**Additional file 1** Supplementary information to GraphAligner: rapid and versatile sequence-to-graph alignment. Details and pseudocode of the *O*(*w*) bitvector merging algorithm and additional figures.

**Additional file 2** Review history.

## Data Availability

Binaries of GraphAligner are available via the package manager Bioconda[66]. The source code of GraphAligner is available on GitHub[67]. The source code of GraphAligner version 1.0.11 used in the experiments is available on Zenodo[68]. Human genome PacBio Sequel data for HG00733 is available from SRA accession SRX4480530 and Illumina from SRA accessions ERR899724, ERR899725, and ERR899726. *D. melanogaster* ONT data is available from SRA accession SRR6702603 and Illumina from SRA accession SRR6702604. *E. coli* PacBio data is available from PacBio at https://github.com/PacificBiosciences/DevNet/wiki/E.-coli-Bacterial-Assemblyand Illumina data from Illumina at ftp://webdata:webdata@ussd-ftp.illumina.com/Data/SequencingRuns/MG1655/MiSeq_Ecoli_MG1655_110721_PF_R1.fastq.gz and ftp://webdata:webdata@ussd-ftp.illumina.com/Data/SequencingRuns/MG1655/MiSeq_Ecoli_MG1655_110721_PF_R2.fastq.gz. Genome in a Bottle variant calls for HG002 are available at ftp://ftp-trace.ncbi.nlm.nih.gov/giab/ftp/release/AshkenazimTrio/HG002_NA24385_son/NISTv3.3.2/GRCh38/. Thousand Genomes variant list is available at http://ftp.1000genomes.ebi.ac.uk/vol1/ftp/data_collections/1000_genomes_project/release/ 20190312_biallelic_SNV_and_INDEL/. Diploid assembly of HG00733 is available at ftp://ftp.1000genomes.ebi.ac.uk/vol1/ftp/data_collections/HGSVC2/working/20200417_Marschall-Eichler_NBT_hap-assm/.

## Footnotes

1 SRA accession SRX4480530

2 SRA accessions ERR899724, ERR899725, ERR899726
