# Supplementary material for: GraphAligner: rapid and versatile sequence-to-graph alignment
Source: Genome Biol. 2020 Sep 24;21:253. doi: 10.1186/s13059-020-02157-2 (PMC7513500; doi:10.1186/s13059-020-02157-2)
Supplement: Supplementary file 2 — Additional file 2 Review history. [file 13059_2020_2157_MOESM2_ESM.docx]

Review History

**First round of review**

**Reviewer 1**

**Were you able to assess all statistics in the manuscript, including the appropriateness of statistical tests used?**

There are no statistics in the manuscript.

**Were you able to directly test the software?**

Yes

**Comments to author:**

The manuscript describes a novel method for aligning long reads to pangenome graphs. The work is cutting-edge as it combines two current topics, the analysis of long read data and the alignment of sequence reads to pan-genome graphs. I am not aware of any other work adressing this problem so far.

I am impressed by the running times of the method. I trust the authors to develop and implement highly useful approaches but, unfortunately, I cannot confirm that their method works well in general based on the little evaluation experiments performed. The results section is very thin giving little evidence on the alignment performance. For publication of a long read pangenome alignment tool in Genome Biology, I would expect a more thorough comparison of alignment quality in addition to alignment speed and quantity. Also, the description of the method lacks formalism and clarity. Overall, my opinion is that this work is not yet ready for publication unless the focus is changed to long read error correction or a considerable amount of alignment quality evaluation is added.

My specific comments are as follows:

- The primary focus of this paper is a "tool for alinging long reads to genome graphs". In the current manuscript, the alignment task is assessed only based on the number of aligned bases, running time and peak memory usage on a single downsampled long read data set. As the number of aligned bases does not necessarily correlate with alignment quality, no evaluation of alignment quality is given. In addition, the downsampled long read data was preselected using minimap2, a standard (= linear genome) long read aligner. This implies that the data set is very simple, where I do not see a need for pangenome graph alignment. Further evaluation, e.g. using simulated data, comparison of alignment positions, evaluation of complex regions, and/or downstream variant calling performance, etc., are needed to show the advantage of pangenome graph alignment.

- The evaluation of error correction performance is well done. It shows a significant improvement in terms of correction performance and running time on the more difficult fly and human genomes compared to other state-of-the-art methods. However, this is only one of many application of long read pangenome graph alignment and the title of the paper promises a "versatile" method. If the claim of versatility was dropped and the focus of the manuscript was shifted to read error correction, I would be convinced by the results.

- Although I am happy to see that many details of the method are mentioned, the Methods section is in many places unclear and needs a thorough revision. Many concepts are described implicitly or ambiguously. The conversion and mapping between bidirected graph and alignment graph is one example, the use of minimizers in chunks is another. I could extend this list by many more examples. In my opinion, the manuscript needs formalism in the description of algorithmic concepts (as given in the author's previous paper, ref. 23, and common in the field).

- I like the idea of the dynamic banding approach but would have liked to see a discussion on how it is different to the X-drop extension used by BLAST (see also Zhang, Berman, [Wiehe], Miller, JCB 1998 / Bioinformatics 1999) and a note on its asymmetry: the band differs if the vertical and horizontal sequences are swapped.

Minor:

- There is some redundancy in the methods section (parallelism is described twice).

- While all other figures are helpful, Figure 8 is not.

- The reference for bidirected graphs should be Edmonds & Johnson, 2003 and not the vg toolkit.

- Both the conda and non-conda install instructions require conda. I chose conda instructions as it is unclear to me how to install without conda.

- Please include a minimal test example in the distribution.

**Reviewer 2**

**Were you able to assess all statistics in the manuscript, including the appropriateness of statistical tests used?**

There are no statistics in the manuscript.

**Were you able to directly test the methods?**

Yes

**Comments to author:**

In this paper Rautiainen and Marschall develop GraphAligner, a new software for aligning long (Pacific Biosciences or Oxford Nanopore) sequencing reads to reference graphs. As the authors note reference graphs are an emerging area of sequence analysis where a traditional linear reference genome is replaced by a richer structure that contains known sequence variation. GraphAligner is one of the first (if not the first) practical software for aligning long reads to a reference graph so I read this paper with interest.

The paper first describes three experiments to demonstrate the performance of GraphAligner relative to other tools in this space. GraphAligner has comparable speed to minimap2 (the most popular long read aligner) when mapping to a linear reference, which is impressive and suggests that its additional flexibility does not have significant overhead when using simpler reference structures. I would have liked to see some quantification of the accuracy of the mappings in this test, for example like Figure 1 of the minimap2 paper.

Next, the authors demonstrate aligning to a reference graph. In this case they augmented human chromosome 22 with known structural variation, and demonstrate that GraphAligner outperforms vg, a general purpose reference graph toolkit. It is not clear to me why SNPs and indels were not added to the reference graph, in addition to SVs. I suspect adding dense SNPs may reduce the performance of the indexing strategy, which is why they only introduced large variation, but it would be good to have an explanation in the text about the choice of variation introduced.

Finally the authors implement a long read error corrector by using GraphAligner to align the long reads to a de Bruijn graph constructed from short reads, and emitting the alignment paths as the corrected sequences. They demonstrate that this approach is an order of magnitude faster, with comparable or better accuracy to competing approaches.

The methods section is clear and contains both algorithmic and engineering details. In the following section, I have questions and comments about some details in the methods.

Specific comments:

1) Page 6: "GraphAligner is presently geared towards aligning long reads, which was our focus due to the absence of methods for this. The current seeding strategy can systematically fail to handle short reads in variation-dense regions."

What is the minimum read length recommended by the authors for use with GraphAligner? This will obviously depend on the density of variation but some guidance for typical (e.g. human) genome graphs would be valuable.

2) Regarding Figure 2, are secondary alignments determined when they overlap on the query (read) or target (reference)?

3) Page 7 states that the input graph can contain overlapping node labels. Do these overlaps need to be exact matches, or can they contain mismatches/insertions/deletions? I suspect the former.

4) One of the key algorithmic advances is the development of the banded dynamic programming described in section 5.6. Recently Suzuki and Kasahara developed a dynamic banding strategy for sequence-to-sequence alignment that uses the scores at the terminal cells of anti-diagonal bands to determine the placement of the next band. I'd be curious to hear the authors' thoughts on this approach and whether it can be applied to sequence-to-graph alignment.

5) I found section 5.8 (Partial Alignments) to be hard to follow, particularly the statements "Given the Viterbi estimate, we define a slice as guaranteed correct as slices where the backtrace for the wrong state is from the correct state". What does "backtrace for the wrong state is from the correct state" mean? Does that mean a "guaranteed correct" slice is one where the inferred state is "correctly aligned" and the state for the next slice is "wrongly aligned"? It would be good if the authors could clarify this section.

**Authors Response**

**Point-by-point responses to the reviewers’ comments:**

Reviewer #1

Comment 1: The manuscript describes a novel method for aligning long reads to pangenome graphs. The work is cutting-edge as it combines two current topics, the analysis of long read data and the alignment of sequence reads to pan-genome graphs. I am not aware of any other work adressing this problem so far.

*Response: We thank the reviewer for appreciating the novelty of our work.*

Comment 2: I am impressed by the running times of the method. I trust the authors to develop and implement highly useful approaches but, unfortunately, I cannot confirm that their method works well in general based on the little evaluation experiments performed. The results section is very thin giving little evidence on the alignment performance. For publication of a long read pangenome alignment tool in Genome Biology, I would expect a more thorough comparison of alignment quality in addition to alignment speed and quantity.

*Response: We agree to the reviewer that an evaluation of alignment quality is important and have now expanded our experiments. We have adopted the evaluation procedure used in the minimap2 paper and show a direct comparison of read mapping accuracy to minimap2, showing that both methods are on par (minimap2: 95.1% accuracy; GraphAligner: 95.0% accuracy). We note that we ran all experiments with the latest version of GraphAligner, where we have added a new seed chaining approach (now described in Methods). This addressed cases of missed alignments we had uncovered in the meantime, but unfortunately also makes GraphAligner slower. It is now slower than minimap2, but still markedly faster even than common linear mappers (like BWA) and by far faster than any other method for sequence-to-graph alignment we are aware of (e.g. about 10x faster than VG and about 12x faster than the error correction method FMRLC). We have re-run all experiments and updated all numbers in the paper. Apart from the slower runtimes and slight changes in numbers here and there, all main conclusions we had presented in our initial submission still apply.*

Comment 3: Also, the description of the method lacks formalism and clarity.

*Response: We reworked the methods part and now describe our approach more formally. In particular, we mathematically define the notion of bidirected graphs and the exact procedure by which we internally translate them into direct graphs for mapping. We have also significantly expanded on the details of the minimizer based seed finding and the newly implemented procedure for seed chaining. We have reworked the corresponding figure (was Figure 5) and now provide two figures, one illustrating the minimizer indexing (Figure 6) and another on “bubble chains” used for seed clustering (Figure 7).*

Comment 4: Overall, my opinion is that this work is not yet ready for publication unless the focus is changed to long read error correction or a considerable amount of alignment quality evaluation is added.

*Response: We have evaluated the alignment quality both when aligning to a linear reference genome, where GraphAligner is approximately as accurate as minimap2 (see response to Comment 2), and to a pangenome graph where GraphAligner is both faster and more accurate than existing tools: When compared to VG, it is about 10x faster and has an accuracy of 96.6%, while VG achieves 93.8%. We note that the VG internally splits long reads and aligns their parts separately, often leading to discontinuous alignments. We show an example of this in Supplementary Figure 2 now. Since our evaluation of accuracy is based on overlap with the true location (inspired by the evaluation in the minimap2 paper), such fragmented alignments are counted as correct even when their utility for downstream applications is limited. Additionally we now provide a new figure (now Figure 1) illustrating the relationship between read length and alignment accuracy.*

*To show that the alignments produced by GraphAligner support applications beyond error correction, we have added a genotyping experiment where we align PacBio HiFi reads of HG002 to a pan-genome graph with GraphAligner and use the alignments to determine the genotypes of variants encoded in bubbles in the graph, achieving F-measures of up to 99.2% for SNPs and 94.4% for indels.*

My specific comments are as follows:

Comment 5: The primary focus of this paper is a "tool for alinging long reads to genome graphs". In the current manuscript, the alignment task is assessed only based on the number of aligned bases, running time and peak memory usage on a single downsampled long read data set. As the number of aligned bases does not necessarily correlate with alignment quality, no evaluation of alignment quality is given. In addition, the downsampled long read data was preselected using minimap2, a standard (= linear genome) long read aligner. This implies that the data set is very simple, where I do not see a need for pangenome graph alignment. Further evaluation, e.g. using simulated data, comparison of alignment positions, evaluation of complex regions, and/or downstream variant calling performance, etc., are needed to show the advantage of pangenome graph alignment.

*Response: As mentioned above, we have added experiments with simulated data evaluating the accuracy of the alignments, showing that the accuracy of GraphAligner is almost identical to the accuracy of minimap2 when mapping to a linear genome (see response to Comment 2), and better than vg when mapping to a variation graph (see response to Comment 4). For these experiments, we have now switched from real long read data to simulated data. On the one hand, this gives us the opportunity to measure mapping accuracy in the first place, and, on the other hand, addresses the reviewers concern that we had preselected read using minimap to align to a linear reference. We have also added an experiment evaluating variant calling using reads mapped to a pangenome with GraphAligner, showing good performance. While we agree with the reviewer that complex variants (such as STRs, VNTRs, paralog-specific variants in segmental duplications, etc.) would be interesting to consider, we note that the full potential of aligning (long and short) reads to a pangenome graph will only become apparent when richer sets of sequence-resolved structural/complex variants are available to construct such graphs. We are actively involved in efforts (such as the Human Pangenome Reference Consortium and the Human Genome Structural Variation Consortium) aiming to generate haplotype resolved assemblies of panels of individual genomes to construct comprehensive pangenome graphs. While GraphAligner is likely to be a key tool once these graphs are available, fully evaluating the performance at complex genomic regions is not yet possible at present in our view. We want to emphasize that we have presented the application of GraphAligner to graphs of drastically varying levels of complexity, ranging from “linear graphs” to de Bruijn graphs of whole human genomes (which tend to be very tangled). We therefore believe that we provide sufficient evidence that GraphAligner performs very favorably across the full spectrum of graph complexities.*

Comment 6: The evaluation of error correction performance is well done. It shows a significant improvement in terms of correction performance and running time on the more difficult fly and human genomes compared to other state-of-the-art methods. However, this is only one of many application of long read pangenome graph alignment and the title of the paper promises a "versatile" method. If the claim of versatility was dropped and the focus of the manuscript was shifted to read error correction, I would be convinced by the results.

*Response: We stand behind our claim that GraphAligner is a versatile tool. Beyond adding the genotyping experiment described above (response to Comment 4), we also now additionally refer to a recent preprint (https://doi.org/10.1101/2020.01.27.921338) where we developed a pipeline that uses GraphAligner to map Oxford Nanopore RNA-seq reads to splice graphs, allowing us to do transcript quantification as well as to detect fusion transcripts. GraphAligner is versatile also with respect to the types of graphs it can work on: In contrast to VG, it can also work with graphs with overlapping node labels. This flexibility enables its applications in many setting, ranging from variation graphs without overlaps to de Bruijn graphs. We now added a sentence emphasizing this aspect to the Conclusions section.*

Comment 7: Although I am happy to see that many details of the method are mentioned, the Methods section is in many places unclear and needs a thorough revision. Many concepts are described implicitly or ambiguously. The conversion and mapping between bidirected graph and alignment graph is one example, the use of minimizers in chunks is another. I could extend this list by many more examples. In my opinion, the manuscript needs formalism in the description of algorithmic concepts (as given in the author's previous paper, ref. 23, and common in the field).

*Response: We thank the reviewer for this suggestion and have reworked the Methods part to address this.*

Comment 8: I like the idea of the dynamic banding approach but would have liked to see a discussion on how it is different to the X-drop extension used by BLAST (see also Zhang, Berman, [Wiehe], Miller, JCB 1998 / Bioinformatics 1999) and a note on its asymmetry: the band differs if the vertical and horizontal sequences are swapped.

*Response: Good point. We now discuss the X-drop extension to the partial alignment section and have added a note about the banding’s asymmetry.*

Comment 9: There is some redundancy in the methods section (parallelism is described twice).

*Response: Thanks. We have removed the redundant section on parallelism.*

Comment 10: While all other figures are helpful, Figure 8 is not.

*Response: We have moved the figure to the supplement. While the figure is not essential to the paper, we believe it can still be useful for readers who are not familiar with the complexity of large de Bruijn graphs.*

Comment 11: The reference for bidirected graphs should be Edmonds & Johnson, 2003 and not the vg toolkit.

*Response: Thanks for pointing this out. We have added the correct reference*

Comment 12: Both the conda and non-conda install instructions require conda. I chose conda instructions as it is unclear to me how to install without conda.

*Response: We have added a list of dependencies to the github readme for compiling without Conda. Still we believe Conda to be the preferred way, given that Conda and Bioconda are rapidly adopted by the bioinformatics community. We also added a note to the readme file to clarify that, when building GraphAligner with the help of Conda, then the Conda environment is only required during build time and not at runtime.*

Comment 13: Please include a minimal test example in the distribution.

*Response: Thanks for the suggestion. We have added a small test example in the github repository.*

Reviewer #2

Comment 14: In this paper Rautiainen and Marschall develop GraphAligner, a new software for aligning long (Pacific Biosciences or Oxford Nanopore) sequencing reads to reference graphs.

As the authors note reference graphs are an emerging area of sequence analysis where a traditional linear reference genome is replaced by a richer structure that contains known sequence variation. GraphAligner is one of the first (if not the first) practical software for aligning long reads to a reference graph so I read this paper with interest.

*Response: We thank the reviewer for this positive assessment.*

Comment 15: The paper first describes three experiments to demonstrate the performance of GraphAligner relative to other tools in this space. GraphAligner has comparable speed to minimap2 (the most popular long read aligner) when mapping to a linear reference, which is impressive and suggests that its additional flexibility does not have significant overhead when using simpler reference structures. I would have liked to see some quantification of the accuracy of the mappings in this test, for example like Figure 1 of the minimap2 paper.

*Response: We thank the reviewer for this suggestion and in particular for pointing us to the evaluation in the minimap2 paper. We have now done this and observe comparable accuracy to minimap2 (95.0% vs. 95.1%), see also our response to Comment 2 of Reviewer 1.*

Comment 16: Next, the authors demonstrate aligning to a reference graph. In this case they augmented human chromosome 22 with known structural variation, and demonstrate that GraphAligner outperforms vg, a general purpose reference graph toolkit. It is not clear to me why SNPs and indels were not added to the reference graph, in addition to SVs. I suspect adding dense SNPs may reduce the performance of the indexing strategy, which is why they only introduced large variation, but it would be good to have an explanation in the text about the choice of variation introduced.

*Response: This is a good point and in fact a high variant density can hamper seed finding. Therefore, we have modified our manuscript to demonstrate that GraphAligner’s seeding strategy still works well when mapping long reads to variation-rich graphs. We now include all variants found on Chromosome 22 by the 1000 Genomes Project, producing a graph with a variant on average every 15 base pairs, and measured alignment accuracy to it along with a comparison of aligning the same reads to a linear reference. To further characterize the settings where we are able to map long reads to such dense graphs, we now provide a new figure (Figure 1) to show the fraction of correctly mapped reads as a function of read length. This analysis reveals that for reads of 1500bp and above, >97% of all reads can be aligned. This is now described in Section 2.2.*

Comment 17: Finally the authors implement a long read error corrector by using GraphAligner to align the long reads to a de Bruijn graph constructed from short reads, and emitting the alignment paths as the corrected sequences. They demonstrate that this approach is an order of magnitude faster, with comparable or better accuracy to competing approaches. The methods section is clear and contains both algorithmic and engineering details. In the following section, I have questions and comments about some details in the methods.

*Response: We thank the reviewer for this positive assessment and answer the remaining points below.*

Comment 18: 1) Page 6: "GraphAligner is presently geared towards aligning long reads, which was our focus due to the absence of methods for this. The current seeding strategy can systematically fail to handle short reads in variation-dense regions."

What is the minimum read length recommended by the authors for use with GraphAligner? This will obviously depend on the density of variation but some guidance for typical (e.g. human) genome graphs would be valuable.

*Response: See our response to Comment 16.*

Comment 19: 2) Regarding Figure 2, are secondary alignments determined when they overlap on the query (read) or target (reference)?

*Response: We have clarified in the text that this is determined from overlaps in the query.*

Comment 20: 3) Page 7 states that the input graph can contain overlapping node labels. Do these overlaps need to be exact matches, or can they contain mismatches/insertions/deletions? I suspect the former.

*Response: Yes, indeed it need to be exact overlaps. We have clarified this in the text.*

Comment 21: 4) One of the key algorithmic advances is the development of the banded dynamic programming described in section 5.6. Recently Suzuki and Kasahara developed a dynamic banding strategy for sequence-to-sequence alignment that uses the scores at the terminal cells of anti-diagonal bands to determine the placement of the next band. I'd be curious to hear the authors' thoughts on this approach and whether it can be applied to sequence-to-graph alignment.

*Response: We have thought about this and did not yet find a promising way to adapt the technique of Suzuki and Kasahara to graphs. We have added a note on this to the banding section (Section 5.7 in Methods).*

Comment 22: 5) I found section 5.8 (Partial Alignments) to be hard to follow, particularly the statements "Given the Viterbi estimate, we define a slice as guaranteed correct as slices where the backtrace for the wrong state is from the correct state". What does "backtrace for the wrong state is from the correct state" mean? Does that mean a "guaranteed correct" slice is one where the inferred state is "correctly aligned" and the state for the next slice is "wrongly aligned"? It would be good if the authors could clarify this section.

*Response: We have edited and amended this section and now include one additional figure (Figure 11) to clarify this.*

**Second round of review**

**Reviewer 1**

I can confirm the author's statement that I am broadly positive about this work. The GraphAligner tool implements a novel approach and the extensive methods section presents many algorithmic tweaks. These are important contributions.

The reviewers have improved the clarity of the methods section significantly. The introduced formalism is not particularly elegant but provides sufficient details. I have only some minor comments left on the methods part (see below).

In my opinion, the evaluation of GraphAligner remains the weakest part of the manuscript:

- In the graph alignment simulation experiment described in Section 2.2, I find the idealized setting unrealistic. I am aware that simulation is always idealized compared to real data. But why not simulate reads from a genome with variants (e.g. obtained with VarSim, doi:org/10.1093/bioinformatics/btu828) to get a little closer to reality?

- My conclusion of the genotyping section (2.4) is that the variant set used to build a variation graph matters and should be chosen with care. This is a nice result but it does not reveal much about GraphAligner's performance. The integration in the vg workflow is an example use case and supports the author's statement on versatility. I would like to know how GraphAligner compares to vg? Is there a reason why you did not compare to genotyping on vg alignments? How does variant calling on a graph compare to variant calling on a linear genome? In terms of variant calling accuracy, is it worth using GraphAligner?

- The comparison to minimap2 in Section 2.1, the comparison to VG in Section 2.3 and the error correction experiment in Section 2.5 are convincing.

Overall, despite the evaluation weaknesses, I am of the opinion that this work should be published.

\### Minor comments:

- Section 2.2 is confusing in terms of reference genome usage. The beginning of the section says GRCh37 was used to build the graph but the second paragraph mentions GRCh38. Also, the middle of the first paragraph says that reads were simulated without more details - were they simulated from the reference genome? If yes, from what version?

- On page 12: B_v1 and B_v2 were not defined. I'd suggest using |sigma_b(v)| instead of the max B_vx.

- Figure 6 is very helpful. Why not flip "k-mer array" and "different k-mer" <- "MPHF" in the "Bucket index 1" box to align it with the text?

- On page 16 sets of good parameters are given. Can you provide an intuition why the different sets are good for dBGs / variation graphs? Don't the parameters used in a dBG depend on the k used for constructing the dBG?

- In algorithm 1, please define/initialize d. Also, I think the d used in Algorithm 1 is different from the d in the text section below. If I am correct with this, I'd suggest replacing one by delta.

\### Typos and language:

- Abstract: I'd suggest replacing "to be over 2x more accurate" by "to be more than twice as accurate".

- Page 3, line 3: "As an another" -> "As another"

- Page 4, first sentence of 2.3: Please revise.

- Several times: "inputed" -> "input" (or at least "inputted")

- Page 13: "leads at least" -> "leads to at least"

**Authors Response**

**Point-by-point responses to the reviewers’ comments:**

Reviewer #1

I can confirm the author's statement that I am broadly positive about this work. The GraphAligner tool implements a novel approach and the extensive methods section presents many algorithmic tweaks. These are important contributions.

*Thank you.*

The reviewers have improved the clarity of the methods section significantly. The introduced formalism is not particularly elegant but provides sufficient details. I have only some minor comments left on the methods part (see below). In my opinion, the evaluation of GraphAligner remains the weakest part of the manuscript:

- In the graph alignment simulation experiment described in Section 2.2, I find the idealized setting unrealistic. I am aware that simulation is always idealized compared to real data. But why not simulate reads from a genome with variants (e.g. obtained with VarSim, doi:org/10.1093/bioinformatics/btu828) to get a little closer to reality?

*We thank the reviewer for this suggestion. Given the recent progresses in de novo genome assembly from PacBio Hifi reads, we decided to use a haplotype-aware assembly of HG00733 (an individual of Puerto Rican descent) as a basis for simulating reads (Porubsky, Ebert, et al., Nature Biotechnology, in press; preprint:* [*https://www.biorxiv.org/content/10.1101/855049v1*](https://www.biorxiv.org/content/10.1101/855049v1)*). Notably, this will include many variants, particularly structural variants, that are not accessible by short read studies. We have added this new experiment to Section 2.2 and Figure 1, as well as to Methods in Section 5.10. While the fraction of mapped reads to the linear reference GRCh37 goes down slidely (as expected; blue curves in Figure 1), the fraction of reads aligned to the variant graph stays virtually identical (red curves in Figure 1). This confirms GraphAligner’s ability to align reads with realistic variant spectra to variation graphs.*

- My conclusion of the genotyping section (2.4) is that the variant set used to build a variation

graph matters and should be chosen with care. This is a nice result but it does not reveal much

about GraphAligner's performance. The integration in the vg workflow is an example use case

and supports the author's statement on versatility. I would like to know how GraphAligner

compares to vg? Is there a reason why you did not compare to genotyping on vg alignments?

*We are glad that this additional section as able to support the versatility of GraphAligner. We have tried to generate vg alignments to perform the suggested comparison, but were not able to use vg to map reads to that the GIAB whole human genome graph that we used in our experiment: When we tried to prune the graph as recommended by the vg documentation, we ran out of memory on a large compute server as vg used more than 1.5 terabytes of memory. When we tried indexing the graph without pruning, we instead ran out of temporary disk space. We hence decided to leave the section as is.*

How does variant calling on a graph compare to variant calling on a linear genome? In terms of variant calling accuracy, is it worth using GraphAligner?

*This is an excellent question that merits in-depth investigation. However, we believe that performing variant calling experiments that do justice to this important question is beyond the scope of this work, especially given that GraphAligner is not a tool for variant calling. Many groups are presently working on tools for variant calling/genotying from reads mapped to genome graphs. Producing accurate sequence-to-graph alignments as input to such methods is crucial, but the variant calling performance will strongly be influenced by such emerging tools. So while we agree that the questions asked by the reviewer are important ones, we feel that it would be premature to attempt to answer them in the frame of this article.*

*How much variant calling will improve upon a linear reference genome will strongly depend on how such graphs are constructed and the largest benefit will become visible when constructing graphs from an extended number of haplotype-resolved de novo genome assemblies (including sequence-resolved structural variants, particularly in repeat contexts such as STRs, VNTRs, MEIs, etc.). We are presently pursuing this direction in the frame of the Human Genome Structural Variation Consortium and the Human Pangenome Reference Consortium, but this is an ongoing process and such comprehensive graphs are not yet available.*

- The comparison to minimap2 in Section 2.1, the comparison to VG in Section 2.3 and the error correction experiment in Section 2.5 are convincing.

*Thank you.*

Overall, despite the evaluation weaknesses, I am of the opinion that this work should be published.

*Thank you.*

\### Minor comments:

- Section 2.2 is confusing in terms of reference genome usage. The beginning of the section says GRCh37 was used to build the graph but the second paragraph mentions GRCh38. Also, the middle of the first paragraph says that reads were simulated without more details - were they simulated from the reference genome? If yes, from what version?

*The mention of GRCh38 was a typo. We have clarified that the reference genome used was GRCh37 and the reads were simulated from GRCh37.*

- On page 12: B_v1 and B_v2 were not defined. I'd suggest using |sigma_b(v)| instead of the max B_vx.

*We have replaced B_v1 and B_v2 with |sigma_b(v1)| and |sigma_b(v2)|.*

- Figure 6 is very helpful. Why not flip "k-mer array" and "different k-mer" <- "MPHF" in the "Bucket index 1" box to align it with the text?

*We have re-organized the bucket index 1 box.*

- On page 16 sets of good parameters are given. Can you provide an intuition why the different sets are good for dBGs / variation graphs? Don't the parameters used in a dBG depend on the k used for constructing the dBG?

*We have added a clarification that the dBG parameters were optimized for k=63, and a brief intuition why different parameters are used for different graphs.*

- In algorithm 1, please define/initialize d. Also, I think the d used in Algorithm 1 is different from the d in the text section below. If I am correct with this, I'd suggest replacing one by delta.

*The d in Algorithm 1 is indeed different from the d in the text section below. We have replaced d in Algorithm 1 with x and added a comment that it is the linearized distance.*

\### Typos and language:

- Abstract: I'd suggest replacing "to be over 2x more accurate" by "to be more than twice as accurate".

- Page 3, line 3: "As an another" -> "As another"

- Page 4, first sentence of 2.3: Please revise.

- Several times: "inputed" -> "input" (or at least "inputted")

- Page 13: "leads at least" -> "leads to at least"

*We have fixed these typos.*

**Third round of review**

**Reviewer 1**

The authors have addressed all remaining points appropriately and I have no further comments.

The added experiment in section 2.2 is well done; it will be interesting to see if graph genome alignment will outperform alignment to linear references in the future.
